# Isomerization of *E*-Cinnamamides into *Z*-Cinnamamides Using a Recycling Photoreactor

**DOI:** 10.1021/acs.joc.4c00721

**Published:** 2024-06-05

**Authors:** Mayuko Suga, Saki Fukushima, Kosho Makino, Kayo Nakamura, Hidetsugu Tabata, Tetsuta Oshitari, Hideaki Natsugari, Noritaka Kuroda, Kunio Kanemaru, Yuji Oda, Hideyo Takahashi

**Affiliations:** † Faculty of Pharmaceutical Sciences, Tokyo University of Science, 2641 Yamazaki, Noda-shi, Chiba 278-8510, Japan; ‡ Research Institute of Pharmaceutical Sciences, Musashino University, Nishitokyo, Tokyo 202-8585, Japan; § Faculty of Pharma Sciences, Teikyo University, 2-11-1 Kaga, Itabashi-ku, Tokyo 173-8605, Japan; ∥ Graduate School of Pharmaceutical Science, The University of Tokyo, 7-3-1 Hongo, Bunkyo-ku, Tokyo 113-0033, Japan; ⊥ YMC Company Limited, 284 Daigo, Karasuma Nishiiru Gojo-dori, Shimogyo-ku, Kyoto 600-8106, Japan; # IWASAKI Electric Company Limited, 1-1, Ichiriyama-cho, Gyoda-shi, Saitama 361-8505, Japan

## Abstract

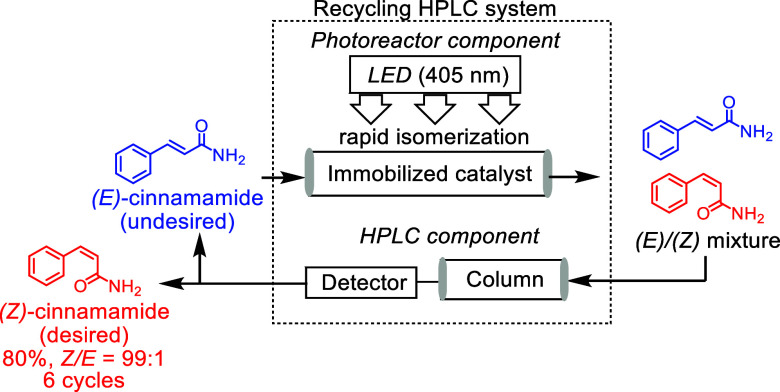

The photocatalytic synthesis of thermodynamically less-stable *Z*-alkenes has received considerable research attention in recent years. In this study, a recycling photoreactor was applied to the photoisomerization of *E*-alkenes (cinnamamide and Weinreb amide derivatives) to produce *Z*-alkenes. The closed-loop recycling system comprises an immobilized photosensitizer to achieve rapid photoisomerization and a high-performance liquid chromatography instrument for separation of the *Z*/*E* diastereomers. After 4–10 cycles, the desired pure *Z*-alkenes were obtained efficiently. In the photoreactor system, a photosensitizer (thioxanthone) was covalently immobilized on silica gel via amide bonding, which led to an enhanced photocatalytic activity compared to the parent thioxanthone. This recycling photoreactor shows promise as an alternative system for the production of *Z*-alkenes.

## Introduction

The photoisomerization of *E*- to *Z-*alkenes finds various applications in organic chemistry, polymer chemistry, and medicinal chemistry since these compounds are ubiquitous structural components in both chemistry and biology.^1^ It is well known that many *Z*-alkenes that cannot be prepared via traditional thermodynamic reactions can be obtained in good yields through photoisomerization. In 2001, Osawa reported the *E*-to-*Z* photoisomerization of 4-cyanostilbene under irradiation with visible light, wherein a ruthenium complex appeared to act as a photosensitizer.^2^ Later, in 2014, the visible-light-mediated *E*-to-*Z* isomerization of allylic amines was achieved in the presence of an iridium photocatalyst.^3^ These contra-thermodynamic isomerization reactions triggered a surge of research into photocatalysis based on energy-transfer (EnT) sensitization.^4^ More specifically, in the photoisomerization of alkenes by EnT catalysis, the main requirement is that the triplet energy of the alkene is below the energy of the photocatalyst. In this context, various iridium- and ruthenium-based polypyridyl complexes are suitable due to their high triplet energies.^5^ Among the economically favorable organic photosensitizers reported to date, Gilmour discovered (−)-riboflavin as a suitable catalyst for the photoisomerization of enone-derived alkenes.^6^

Inspired by these previous results, our group explored a number of organic catalysts in the *E*-to-*Z* photoisomerization of alkenes, with the aim of applying the most favorable system in a recycling photoreactor. In our previous paper,^7^ a recycle photoreactor was developed based on the deracemization concept,^8^ wherein a racemate is converted into a pure enantiomer. Applying this system to the synthesis of chiral alkyl aryl sulfoxides, the desired pure chiral sulfoxides were efficiently obtained after 4–6 cycles. In the context of a continuous-flow system, Rueping achieved an efficient *E*-to-*Z* photoisomerization of alkenes in which the photocatalyst was immobilized in an ionic liquid and was continuously recycled via a simple phase separation process.^9^ However, current methods based on the use of ionic liquids are time-consuming and are difficult to apply to the recycling high-performance liquid chromatography (HPLC) technology.

Thus, in the present study, the photoisomerization of *E*- to *Z*-alkenes is carried out using a recycling photoreactor coupled with an HPLC system. In addition, the catalytic efficiency of the immobilized photosensitizer is compared with that of the nonimmobilized parent photosensitizer.

## Results and Discussion

### Preparation of the Immobilized Photosensitizer

To realize the *E*-to-*Z* photoisomerization of alkenes in the photoreactor containing a recycling HPLC system, it is necessary to employ a photosensitizer that promotes rapid photoisomerization. Thus, a range of widely used and commercially available photosensitizers (**A–F**) were evaluated for the photoisomerization of *E*-cinnamamide **1a** in acetonitrile (MeCN). In this experiment, each photosensitizer was irradiated with light corresponding to its maximum absorption wavelength. The diastereomeric ratio achieved in each reaction was determined using ^1^H nuclear magnetic resonance (NMR) spectroscopy, and the results are presented in Table 1. More specifically, upon the irradiation of a 20 mM solution of *E*-**1a** using a light-emitting diode (LED; λ = 425 nm) for 15 min, no reaction was observed in the presence of 2,4,6-triphenylpyrylium tetrafluoroborate (**A**) or 9,10-dicyanoanthracene (**B**). Similarly, Mes-Acr-Me^+^ (**C**) was also ineffective and was found to decompose during the reaction. However, using 5 mol% anthracene (**D**) or xanthone (**E**), LED irradiation at λ = 365 nm induced slight isomerization. Notably, thioxanthone (**F**) gave the most desirable result, with ^1^H NMR analysis confirming the generation of a 47:53 *Z*/*E* mixture of cinnamamide **1a** (see Supporting Information Figure S1).

**Table 1:**
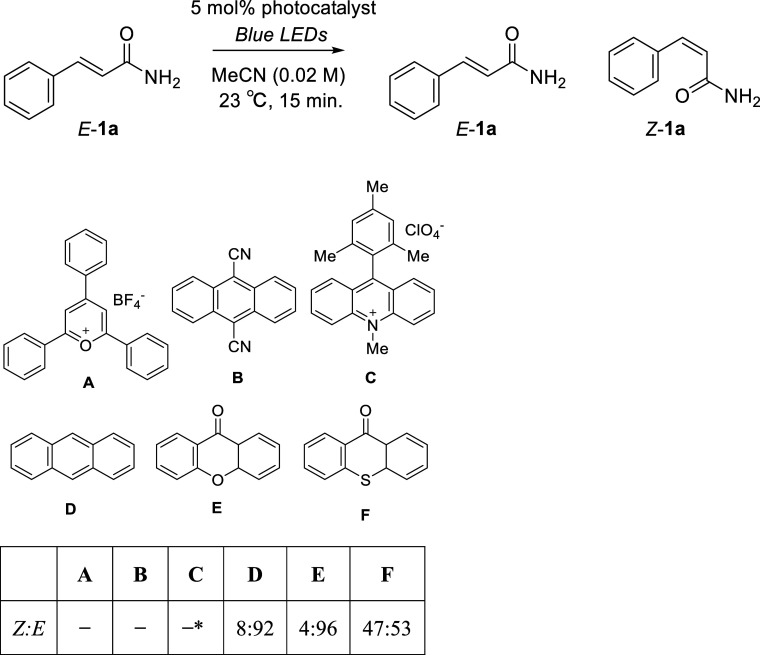
Photocatalyst Screening for the Isomerization of **1a**

a Decomposed.

With the optimal photosensitizer in hand, its immobilization on a solid phase was subsequently investigated. To prevent catalyst leaching, covalent bonding appears to be the most effective approach.^9,10^ Thus, for the purpose of this study, 3-aminopropyl silica gel was employed as a solid support due to its nucleophilic nature that allows it to partake in covalent bonding. However, to successfully immobilize thioxanthone on the solid support, it was first necessary to introduce suitable functional groups into the thioxanthone structure.

**Scheme 1:**
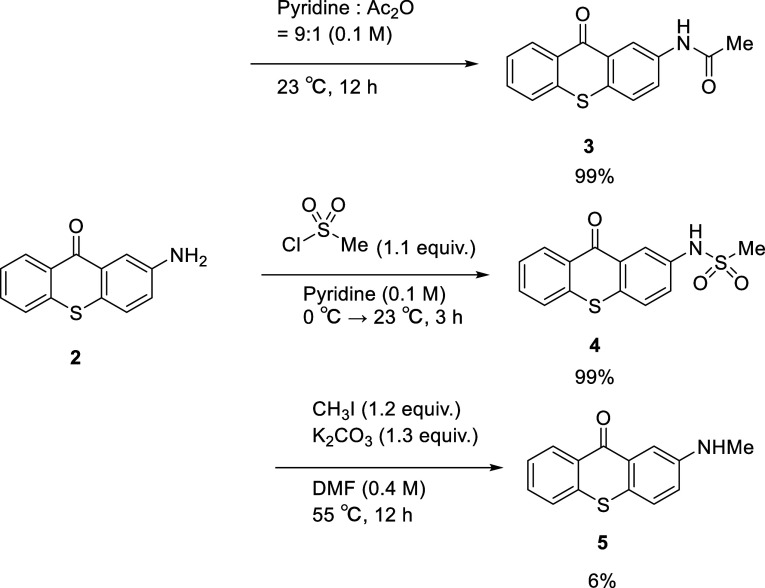
Introduction of Functional Groups into the Thioxanthone Structure

2-Aminothioxanthone **2**, which was prepared according to a previously reported procedure,^11^ was converted to acetamide **3**(12) and methylsulfonamide **4** quantitatively (Scheme 1). However, the direct methylation of **2** provided methylamine **5**(11) in a poor yield. To clarify whether the introduced substituents affect the catalytic activity of thioxanthone, it was evaluated along with its derivatives (**2**–**5**) using the model reaction, namely the photoisomerization of *E*-cinnamamide **1a** (*E*-**1a**). Upon the irradiation of a 10 mM solution of *E*-**1a** using LED light (λ = 405 nm) in the presence of the various photosensitizers (5 mol%, thioxanthone and compounds **2**–**5**), changes in the ratio of *E*-**1a** were determined by HPLC. The originally developed photoreaction evaluation device was employed for this purpose, which enables strict control of the irradiance dose (i.e., distribution, temperature, and time; see Figure S2). When carrying out a photoreaction, it is important to measure the amount of light irradiation required to promote the reaction,^13^ and this device renders it possible to measure how the reaction proceeds in response to the total irradiance dose. The catalytic activities of the various photosensitizers were calculated by comparing the total amount of light irradiation required to reach a certain *Z*/*E* ratio, wherein a more active catalyst requires a smaller amount of light irradiation. In the presence of compounds **2** and **5**, no significant isomerization of *E*-**1a** was observed, indicating that they were less effective than thioxanthone. However, in the presence of compounds **3** and **4**, the isomerization reaction proceeded more rapidly than when thioxanthone was employed (Figures S3 and S4; Table S1).

Based on the data presented in Figure S3, the integrated irradiance required to achieve 55% isomerization of *E*-**1a** was calculated (see Figure S3, Table S1, and Table 2). It was found that compound **3** required the least amount of irradiation (Table 2, entry 3), and this catalyst was found to be four times more active than thioxanthone. It was reasoned that the introduction of an amide group into the thioxanthone skeleton increased the catalytic activity. In 2020, Elliott and Booker-Milburn reported that the introduction of auxochromes into the thioxanthone core enabled fine-tuning of the UV–vis absorption properties and their associated triplet energies, which are known to affect the efficacy of a photosensitizer.^14^ With this in mind, the UV–vis absorption properties of thioxanthone, **2**, and **3** were evaluated, and their corresponding maximum absorption wavelengths were defined as 382, 426, and 394 nm, respectively (Figure S5). Based on these results, catalyst **3** was identified as the optimal catalyst for this reaction; this was accounted for by the fact that its maximum absorption wavelength is the closest to the irradiated wavelength (405 nm) employed herein.

**Table 2:**
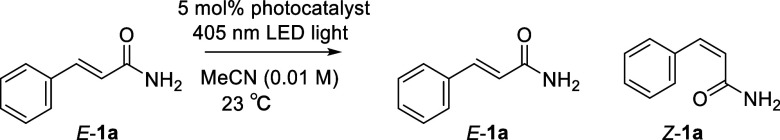
Integrated Irradiance (mJ/cm^2^) Required for the Isomerization of *E*-**1a**

entry	compound	integrated irradiance (mJ/cm^2^)
1	thioxanthone	1.66 × 10^4^
2	**2**	1.50 × 10^5^
3	**3**	4.13 × 10^3^
4	**4**	5.78 × 10^3^
5	**5**	1.49 × 10^5^
6	**7a**	4.38 × 10^3^
7	**7c**	5.16 × 10^3^
8	**7e**	6.70 × 10^3^

**Scheme 2:**
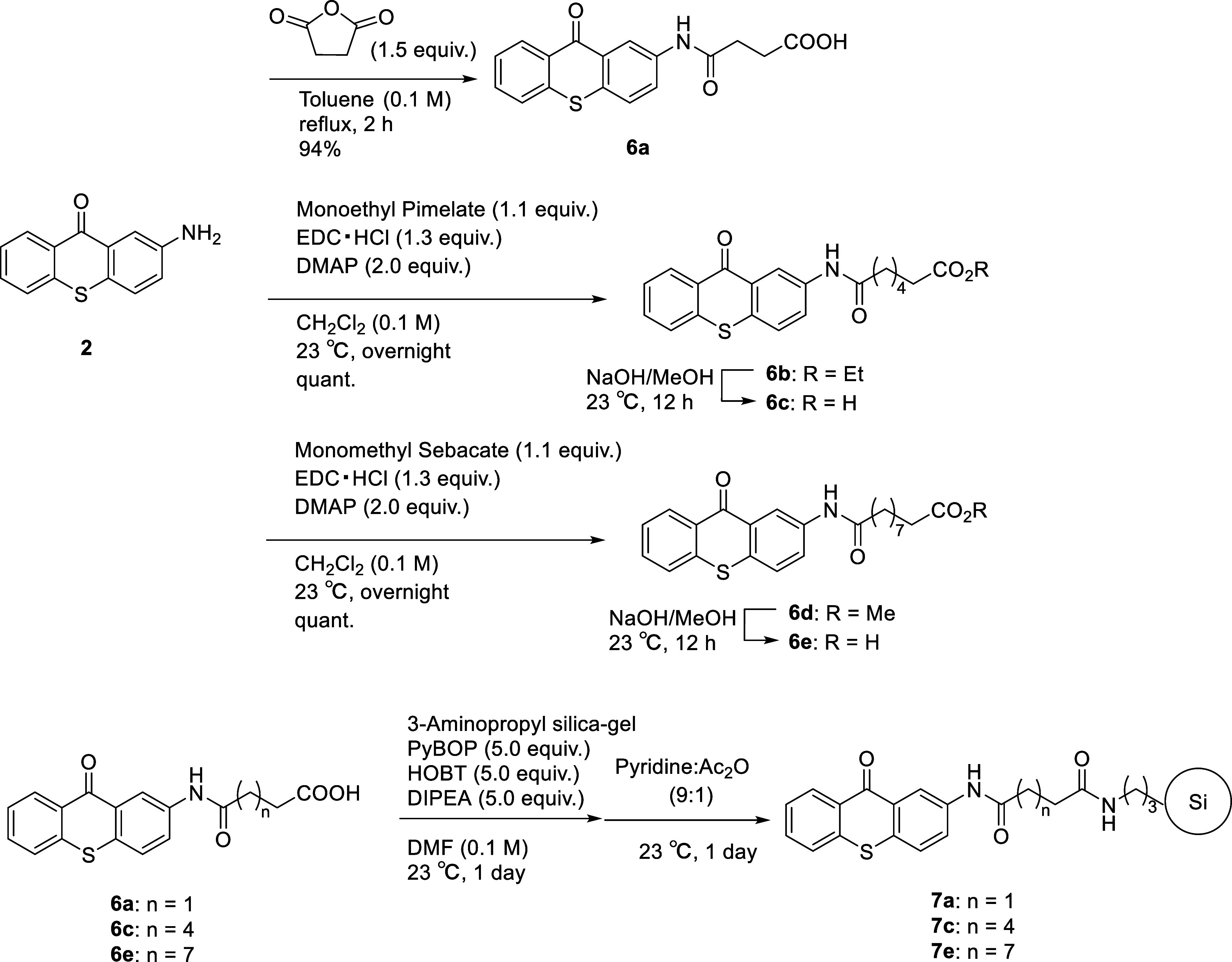
Preparation of the Supported Photosensitizer

Thus, with the optimal photosensitizer in hand, the amide bond was selected for linkage to the solid 3-aminopropyl silica gel support. The attachment of thioxanthone to the support was achieved by a process involving the treatment of **2** with succinic anhydride to provide compound **6a**, containing an amide-bonded tether bearing an end-chain carboxy group. Similarly, compound **2** was coupled with monoethyl pimelate/monomethyl sebacate via a carbodiimide-based activation protocol to provide **6b**/**6d**, respectively. Subsequent hydrolysis of these compounds converted their ester groups into carboxylic acids (compounds **6c** and **6e**). The terminal carboxyl groups of **6a**, **6c**, and **6e** were then condensed with the amino group of the 3-aminopropyl silica gel in the presence of PyBop (1*H*-benzotriazol-1-yloxytripyrrolidinophosphonium hexafluorophosphate) and HOBt (1-hydroxybenzotriazole) to form stable covalent amide bonds and yield compounds **7a**, **7c**, and **7e** (Scheme 2). Following completion of the immobilization reaction, any remaining unreacted amine groups on the support were acetylated using a large excess of acetic anhydride and pyridine.^15^

Using the above-described photoreaction device, the catalytic activities of **7a**, **7c**, and **7e** were evaluated in the photoisomerization of *E*-**1a**. Upon the irradiation of a 10 mM solution of *E*-**1a** using LED light (λ = 405 nm) in the presence of 5 mol% **7a**, **7c**, and **7e**, changes in the ratio of *E*-**1a** were measured by HPLC (Figure S6). The integrated irradiances required to achieve 55% isomerization were then calculated (Table 2, entries 6–8, Table S2), and it was found that all solid-supported catalysts (i.e., **7a**, **7c**, and **7e**) required less irradiation than the soluble parent thioxanthone. This result is particularly interesting due to the fact that solid-phase reactions are generally slower than liquid-phase reactions, whereas the opposite was true here.

The most active catalyst, **7a**, which is 3.8 times more active than thioxanthone, was subsequently employed as the optimal solid-support catalyst to evaluate the possibility of thioxanthone leakage from the immobilized catalyst during the model reaction. After stirring for 10 min in MeCN, the solid catalyst (**7a**) was separated by filtration, and the filtrate was employed in the photoisomerization of *E*-**1a**. Notably, the reaction failed to take place under these conditions, thereby indicating that thioxanthone leaching did not occur (Figure S7).

Moreover, recycling studies of **7a** were conducted using the model reaction. The reaction conditions were the same as those described above, and the progress of each run was determined by the *Z*/*E* ratio **1a**. After completion of each run (30 min), the solid catalyst was separated by filtration, washed successively with CH_3_CN and CH_2_Cl_2_, dried, and then reused in the subsequent run. **7a** was reused up to 10 times, with the reaction rate remaining almost unchanged (Supporting Information).

### Isomerization of *E*- to *Z*-Alkenes Using the Recycling Photoreactor

**Figure 1:**
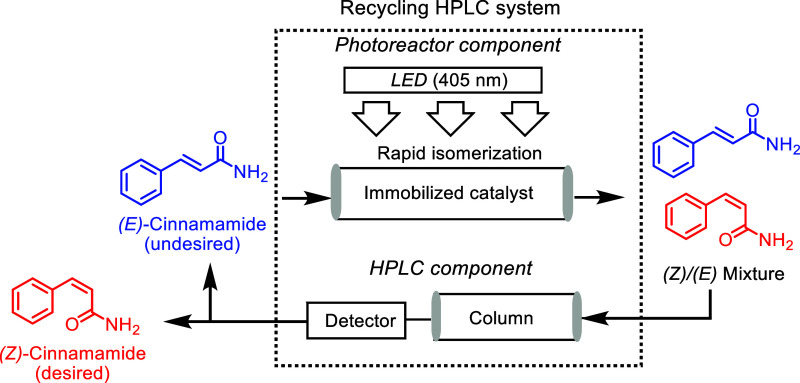
General flowchart representing the recycling photoreactor.

The recycling photoreactor system comprises a photoreactor (to enable rapid isomerization) and an HPLC instrument (for diastereomer separation), as shown in Figure S8. To achieve successful isomerization using this system, rapid photoisomerization should be conducted on the solid phase under continuous flow conditions. In this photoreactor, the immobilized catalyst was packed into a glass tube, which was covered with a device that irradiated LED light. In the photoreactor component, increasing light transmission to the central region of the glass tube is essential. Thus, light-permeable glass beads, which possess a comparable particle radius to the catalyst, were employed to yield a more efficient light distribution. Based on the results of our previous paper, in which 2 wt% of the solid catalyst was used,^7^ 5 wt% **7a** was used due to the fact that it exhibited the highest catalytic efficiency. Thus, **7a** was packed into a glass tube, and the prepared photoreactor was incorporated into the recycling HPLC system for isomerization of the *E*-alkene under light irradiation conditions (Figure 1).

**Figure 2:**
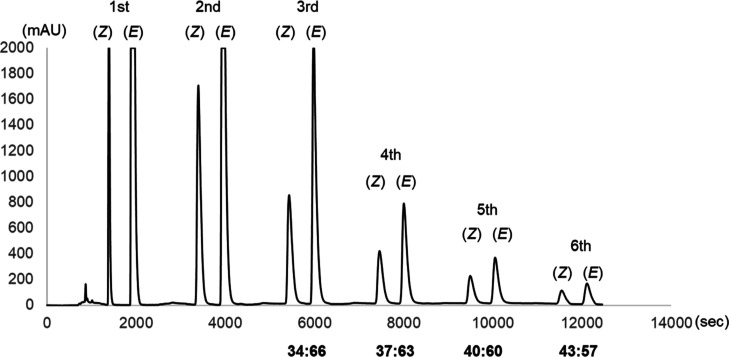
Diastereomeric enrichment of *Z*-**1a** in the recycling photoreactor. Column: YMC-Pack SIL-06 (ϕ: 20 mm, length: 250 mm), mobile phase: CH_3_CN, flow rate: 4.7 mL/min. The fractions containing the *Z*-isomer (*Z*) were collected and accumulated under continuous flow conditions.

Prior to carrying out the photoisomerization reaction, the *Z*/*E* ratio of cinnamamide **1a** was examined at equilibrium. More specifically, a 10 mM solution of *E*-**1a** was irradiated (405 nm) in the presence of the immobilized catalyst (**7a**, 5 mg) for 15 min, and the reaction progress was monitored by HPLC. At this point, the isomerization was confirmed to have reached an equilibrium state (*Z*/*E* = ∼60/40) (Figure S9). Notably, if only *Z*-**1a**^5b^ is removed from this system, the equilibrium state will continue to be biased toward *Z*-**1a**, as embodied by the closed-loop recycling of the photoreactor employed herein. Thus, *E*-**1a** (10 mg) was injected into the recycling photoreactor system and flowed through the reactor (ϕ: 5 mm, length: 21 cm) at a rate of 4.7 mL/min under optical irradiation (405 nm). Consequently, the expected photoisomerization occurred, and the obtained *Z*/*E*-mixture of **1a** was determined by HPLC (YMC-Pack SIL-06 solid phase) to be 25:75 (Figure 2, first run). This result indicates that incomplete isomerization was achieved in the first run, and so despite the suitability of the immobilized catalyst, its catalytic activity appeared too slow for application in this system. Compared to our previously reported immobilized catalyst (*k*_obs_ = 1.99 × 10^–2^ M^–1^ s^–1^),^7^ which was used for the racemization of chiral sulfoxides in a photoreactor, the catalytic activity of **7a** (*k*_obs_ = 9.1 × 10^–3^ M^–1^ s^–1^) was indeed lower. Thus, the desired *Z*-**1a** fraction was collected, and the undesired *E*-**1a** fraction was recycled and flowed through the photoreactor once again. After the desired irradiation time, the isomerized *Z*-**1a** was again separated by HPLC, and the unreacted *E*-**1a** was subjected to a subsequent isomerization cycle. After the third, fourth, fifth, and sixth runs, the obtained *E*-**1a**:*Z*-**1a** ratios were determined to be 34:66, 37:63, 40:60, 43:57, respectively (Figure 2), indicating that the *Z*-**1a** fraction gradually increased during the later cycle runs. This was attributed to the fact that lower quantities of *E*-**1a** were present in the later cycles, and so isomerization proceeded more quickly. After the sixth run, the *Z*-**1a** components accumulated over six cycles gave a yield of 80% with a *Z*/*E* ratio of 99:1 (Table 3, entry 1; Figures S12 and S13).

**Table 3:**
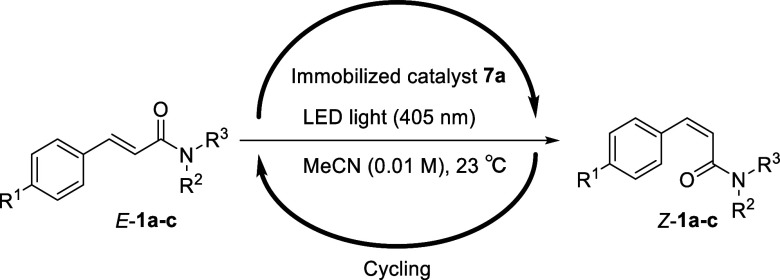
Scope of the Reaction^a^

entry	compound	R^1^	R^2^	R^3^	number of cycles	yield (%)	*Z*/*E* ratio
1	*E*-**1a**	H	H	H	6	80	99:1
2^b^	*E*-**1b**^16^	OMe	Me	OMe	4	70	98:2
3	*E*-**1b**^16^	OMe	Me	OMe	9	64	>99:1
4	*E*-**1c**^16^	H	Me	OMe	10	68	>99:1

a The HPLC conditions and the corresponding chromatograms are provided in the Supporting Information.

b 4 mg of **1b** was used.

**Figure 3:**
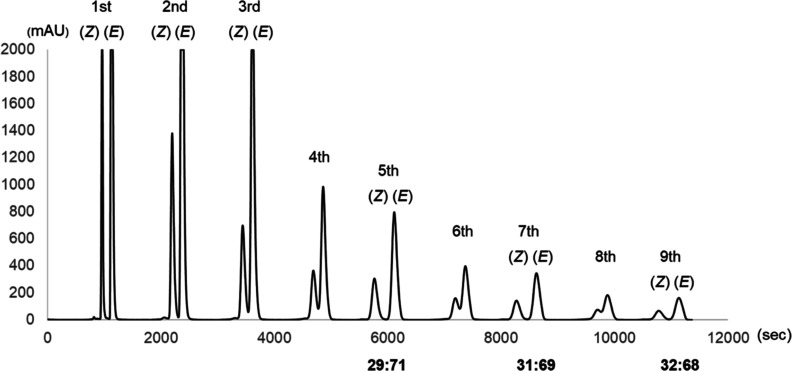
Diastereomeric enrichment of *Z*-**1b** in the recycle photoreactor. Column: YMC-Pack SIL-06 (ϕ: 20 mm, length: 250 mm), mobile phase: CH_3_CN, flow rate: 4.7 mL/min. The fractions containing the *Z*-isomer (*Z*) were collected and accumulated under continuous flow conditions.

Subsequently, Weinreb amides *E*-**1b** and *E*-**1c**, which are versatile intermediates that can be easily converted to aldehydes and ketones,^17^ were subjected to isomerization in the recycling photoreactor. Prior to carrying out the isomerization of these compounds, their *Z*/*E* ratios were determined at equilibrium. After 15 min of irradiation under LED light (405 nm), ratios of 72:28 and 78:22 were determined for **1b** and **1c** (Figures S10 and S11). Thus, *E*-**1b** (4 mg) was injected into the recycle photoreactor system and flowed at a rate of 4.7 mL/min through the photoreactor (ϕ: 5 mm, length: 21 cm) under optical irradiation (405 nm). After four cycles, a 70% yield of *Z*-**1b** was obtained with a *Z*/*E* ratio of 98:2 (Table 3, entry 2; Figures S14 and S15). As in the case of **1a**, the *Z*-**1b** peaks were found to increase gradually in intensity during the later cycle runs. To examine the possibility of isomerizing larger quantities of compounds, 10 mg of *E*-**1b** was injected into the recycling photoreactor system under identical conditions. However, the YMC-Pack SIL-06 column was unable to effectively separate the *Z*/*E*-diastereomers of **1b** and, as a result, the gradual peak broadening during later runs became an obstacle to collecting *Z*-**1b** (Table 3, entry 3; Figure 3). To address this issue, in the fourth cycle, *Z*-**1b** was passed through the column without prior isolation, and after the fifth cycle, the generated *Z*-**1b** was isolated. Similarly, *Z*-**1b** was passed through the column without separation in the sixth and eighth cycles. After the ninth run, the desired *Z*-**1b** was accumulated in a yield of 64% with a *Z*/*E* ratio of >99:1 (Table 3, entry 3; Figures S16 and S17). Finally, 10 mg of *E*-**1c** was injected into the recycling photoreactor. Consequently, *Z*-**1c**^18^ was accumulated in a similar manner to **1b** over 10 cycles, giving a yield of 68% with a *Z*/*E* ratio of >99:1 (Table 3, entry 4; Figures S18 and S19).

## Conclusions

A recycling photoreactor that comprises a rapid photoisomerization chamber and an HPLC system for diastereomer separation was reported to obtain thermodynamically less-stable *Z*-cinnamamide and *Z*-Weinreb amide derivatives.^19^ This process was based on the rapid photoisomerization of the corresponding *E-*alkenes in the presence of an immobilized photosensitizer, namely thioxanthone. The immobilization of thioxanthone on modified silica gel through covalent amide bonds prevented leakage of the photosensitizer from the solid phase, while also enhancing its catalytic activity compared to the soluble parent thioxanthone. Considering that solid-phase reactions are generally slower than liquid-phase reactions, this improvement in the catalytic activity of thioxanthone is particularly interesting. It was reasoned that the introduction of a suitable functional group was responsible for this superior catalytic activity. The catalytic efficiencies of various photosensitizers bearing different functional groups were therefore estimated by comparing the total amount of light irradiation required to promote the photoisomerization reaction, and the optimal system was identified. After 4–10 cycles, the desired pure *Z*-alkenes were obtained in good yields. Overall, this recycling photoreactor shows promise as an alternative system for the production of *Z*-alkenes. However, peak broadening during later cycle runs complicated the separation process, and so the accumulated yields after several cycles were lower than expected. In future work, it would be interesting to employ a twin-column multicolumn countercurrent solvent gradient purification process to enable internal recycling of the unseparated eluting stream. Moreover, careful selection of HPLC conditions is required, since the HPLC solvent is determined based on the photoisomerization solvent since a closed-loop recycling system is used. To address this issue, photoisomerization reactions using mixed solvents that are suitable for HPLC usage are currently being investigated in our laboratory, and the results will be reported in due course.

## Experimental Section

### General Information

All reagents were purchased from commercial suppliers and were used as received. The reaction mixtures were stirred magnetically and were monitored using thin-layer chromatography on precoated silica gel plates. An oil bath was used for all reactions that required heating. Column chromatography was performed using silica gel (45–60 μm), and all extracted solutions were dried over anhydrous Na_2_SO_4_. After filtration, the solvents were evaporated under reduced pressure, and NMR spectra were recorded at 400 MHz, 600 MHz for ^1^H NMR, and at 100 MHz for ^13^C NMR, respectively, at 293 K, unless otherwise stated. Tetramethylsilane (TMS, δ 0.00) and residual internal CHCl_3_ (^1^H NMR: δ 7.26 and ^13^C NMR: δ 77.16) were used as the internal references for the ^1^H and ^13^C NMR spectra of the samples run in CDCl_3_. The coupling constants (*J*) are reported in Hertz (Hz), and the splitting patterns are abbreviated as follows: singlet (s), doublet (d), triplet (t), quartet (q), multiplet (m), and broad (br). Structural assignments were made with additional information from COSY, HSQC, and HMBC experiments. High-resolution mass spectrometry (MS) was conducted using the electrospray ionization time-of-flight (TOF), atmospheric pressure chemical ionization-TOF, and electron impact mass spectrometry techniques. The Fourier transform infrared (FTIR) spectra were recorded in the attenuated total reflectance mode (diamond). The melting points (mps), which are uncorrected, were recorded using a melting point apparatus. An optical irradiation device (Evoluchem PhotoRedOx Box) and chemistry screening kits (HepatoChem Inc., MA, USA) were used for LED irradiation.

### General Experimental Procedures

#### *Z*-**1b**:

Commercially available *E*-**1b** was subjected to photoisomerization to yield mixtures of *E*-**1b** and *Z*-**1b**, from which *Z*-**1b** was isolated with >99% purity by means of HPLC. The chromatographic conditions were as follows: Column, YMC-Pack SIL-06: YMC Co., Ltd. Kyoto; eluent, 100% MeCN; flow rate, 0.5 mL/min; temperature, 25 °C; Rt, 12 min for *Z*-**1b** and 14 min for *E*-**1b**. The product was obtained as a colorless oil. IR (ATR): 1645, 1603 cm^–1^. ^1^H NMR (CDCl_3_, 600 MHz): δ 7.55 (br s, 2H), 6.87–6.84 (m, 2H), 6.70 (brd, 1H, *J* = 12.8 Hz), 6.19 (br s, 1H), 3.81 (s, 3H), 3.68 (br s, 3H), 3.25 (br s, 3H). ^13^C{^1^H} NMR (CDCl_3_, 100 MHz): δ 160.0, 131.2, 127.9, 113.7, 55.3. (Several signals broaden) HRMS (ESI-TOF) *m*/*z*: [M + H]^+^ calcd for C_12_H_16_NO_3_, 222.1130; found, 222.1129.

#### Synthesis of **3**(12):

Compound **2** (51 mg, 0.224 mmol) was added to a solution of pyridine/acetic anhydride = 9:1 (2 mL, 0.1 M), and the mixture was stirred at room temperature for 12 h. After this time, the reaction was quenched with water (10 mL) and extracted using ethyl acetate (EtOAc, 100 mL). The organic extract was washed with brine, dried (anhydrous Na_2_SO_4_), filtered, and concentrated under reduced pressure. The residue was purified by column chromatography on silica gel using hexane/EtOAc (2:1 v/v) to afford **3** (54 mg, 0.222 mmol, 99%) as a yellow solid. mp 243–245 °C.

#### Synthesis of **4**:

Compound **2** (53 mg, 0.233 mmol) was added to a stirred solution of methanesulfonyl chloride (0.043 mL, 0.256 mmol, 1.1 equiv) in pyridine (2.5 mL, 0.1 M) at 0 °C. After stirring at room temperature for 3 h, the mixture was quenched with MeOH and concentrated under reduced pressure. The residue was purified by column chromatography on silica gel using hexane/EtOAc (1:1 v/v) to afford **4** as a yellow solid (70.4 mg, 99%). mp: 261–263 °C. IR (ATR): 3346, 1977 cm^–1^. ^1^H NMR (DMSO-*d*_6_, 400 MHz): δ 10.24 (br s, 1H), 8.46 (dd, 1H, *J* = 8.4, 1.2 Hz), 8.32 (d, 1H, *J* = 2.4 Hz), 7.86–7.82 (m, 2H), 7.79–7.74 (m, 1H), 7.65 (dd, 1H, *J* = 8.4, 2.4 Hz), 7.60–7.55 (m, 1H), 3.06 (s, 3H). ^13^C{^1^H} NMR (DMSO-*d*_6_, 100 MHz): δ 178.5, 137.6, 136.6, 133.0, 131.3, 129.1, 127.9, 126.8, 126.6, 125.2, 118.4. (Several signals overlapped) HRMS (ESI-TOF) *m*/*z*: [M + H]^+^ calcd for C_14_H_12_NO_3_S_2_, 306.0253; found, 306.0253.

#### Synthesis of **5**(11):

A solution of **2** (51 mg, 0.224 mmol), potassium carbonate (40 mg, 0.292 mmol, 1.3 equiv), methyl iodide (0.017 mL, 0.269 mmol, 1.2 equiv), and DMF (0.5 mL, 0.4 M) was stirred for 12 h at 55 °C. After this time, the reaction was quenched with water and extracted with EtOAc (100 mL). The organic extract was washed with brine, dried (anhydrous Na_2_SO_4_), filtered, and concentrated under reduced pressure. The residue was purified by column chromatography on silica gel using hexane/EtOAc (2:1 v/v) to afford **5** as a yellow solid (3.2 mg, 0.0134 mmol, 6%). mp 150–152 °C.

#### Synthesis of **6a**:

A mixture of **2** (504 mg, 2.22 mmol) and succinic anhydride (334 mg, 3.33 mmol, 1.5 equiv) in toluene (23 mL, 0.1 M) was stirred at 120 °C for 2 h. After this time, the reaction mixture was filtered and the residue was dried in vacuo overnight to afford **6a** as a yellow solid (682 mg, 94%). mp 207–208 °C. IR (ATR): 3196, 2028, 1977 cm^–1^. ^1^H NMR (DMSO-*d*_6_, 400 MHz): δ 12.17 (br s, 1H), 10.36 (s, 1H), 8.72 (d, 1H, *J* = 2.4 Hz), 8.46 (dd, 1H, *J* = 1.2, 8.8 Hz), 8.01 (dd, 1H, *J* = 2.4, 8.8 Hz) 7.83–7.73 (m, 3H), 7.59–7.54 (m, 1H), 2.64–2.53 (m, 4H). ^13^C{^1^H} NMR (DMSO-*d*_6_, 100 MHz): δ 178.6, 173.8, 170.6, 138.2, 132.8, 130.2, 129.1, 128.9, 128.0, 127.1, 126.6, 126.5, 124.5, 117.9, 31.1, 28.7. (Several signals overlapped) HRMS (ESI-TOF) *m*/*z*: [M + H]^+^ calcd for C_17_H_14_NO_4_S, 328.0638; found, 328.0633.

#### Synthesis of **6b**:

A mixture of **2** (50 mg, 0.220 mmol), monoethyl pimelate (45.5 mg, 0.242 mmol, 1.1 equiv), 1-(3-dimethylaminopropyl)-3-ethylcarbodiimide hydrochloride (43.3 mg, 0.226 mmol, 1.3 equiv), and 4-dimethylaminopyridine (42.4 mg, 0.347 mmol, 2.0 equiv) in CH_2_Cl_2_ (2 mL, 0.1 M) was stirred at 23 °C for 15 h. After this time, the reaction was quenched with water and extracted with CH_2_Cl_2_ (100 mL). The organic extract was washed with brine, dried (anhydrous Na_2_SO_4_), filtered, and concentrated under reduced pressure. The residue was purified by column chromatography on silica gel using CH_2_Cl_2_/MeOH (14:1 v/v) as the eluent to afford **6b** as a yellow solid (85.7 mg, 98%). mp 124–126 °C. IR (ATR): 3339, 1727, 1696 cm^–1^. ^1^H NMR (CDCl_3_, 400 MHz): δ 8.60 (dd, 1H, *J* = 0.8, 8.0 Hz), 8.43 (dd, 1H, *J* = 2.4, 8.8 Hz), 8.35 (d, 1H, *J* = 2.8 Hz), 7.89 (br s, 1H), 7.65–7.56 (m, 3H), 7.51–7.46 (m, 1H), 4.14 (q, 2H, *J* = 7.2 Hz), 2.44 (t, 2H, *J* = 7.2 Hz), 2.33 (t, 3H, *J* = 7.2 Hz), 1.81–1.73 (m, 2H), 1.72–1.64 (m, 2H), 1.47–1.39 (m, 2H), 1.25 (t, 3H, *J* = 7.2 Hz). ^13^C{^1^H} NMR (CDCl_3_, 100 MHz): δ 179.9, 173.8, 171.8, 137.7, 137.3, 132.3, 132.2, 129.7, 129.3, 128.6, 126.9, 126.2, 126.1, 125.5, 119.2, 60.4, 37.3, 34.0, 28.6, 25.1, 24.5, 14.3. HRMS (ESI-TOF) *m*/*z*: [M + Na]^+^ calcd for C_22_H_23_NNaO_4_S, 420.1245; found, 420.1245.

#### Synthesis of **6c**:

Ester **6b** (50 mg, 0.126 mmol) was hydrolyzed with NaOH (51 mg, 1.26 mmol, 10 equiv) in MeOH (2.0 mL, 0.05 M) at 23 °C for 15 h. After this time, the reaction was quenched with a 2 N aqueous HCl solution and extracted with EtOAc (100 mL). The organic extract was washed with brine, dried (anhydrous Na_2_SO_4_), filtered, and concentrated under reduced pressure. The residue was purified by column chromatography on silica gel using CH_2_Cl_2_/MeOH (9:1 v/v) to give **6c** as a yellow solid. (46.5 mg, 98%). mp 215–216 °C. IR (ATR): 3275, 1693, 1636 cm^–1^. ^1^H NMR (DMSO-*d*_6_, 400 MHz): δ 10.29 (br s, 1H), 8.72 (d, 1H, *J* = 2.4 Hz), 8.46 (dd, 1H, *J* = 1.2, 8.0 Hz), 8.04 (dd, 1H, *J* = 2.4, 8.4 Hz), 7.84–7.73 (m, 3H), 7.60–7.55 (m, 1H), 2.34 (t, 2H, *J* = 7.2 Hz), 2.20 (t, 2H, *J* = 7.2 Hz), 1.65–1.57 (m, 2H), 1.56–1.48 (m, 2H), 1.36–1.27 (m, 2H). ^13^C{^1^H} NMR (DMSO-*d*_6_, 400 MHz): δ 179.2, 175.1, 172.1, 138.8, 137.2, 133.3, 130.7, 129.7, 129.3, 128.5, 127.6, 127.1, 127.0, 125.1, 118.5, 36.8, 34.2, 28.8, 25.3, 24.9. HRMS (ESI-TOF) *m*/*z*: [M + Na]^+^ calcd for C_20_H_19_NNaO_4_S, 392.0932; found, 392.0932.

#### Synthesis of **6d**:

Compound **6d** was prepared from compound **2** (39.4 mg, 0.174 mmol) and monomethyl sebacate according to the procedure described for compound **6b**, and the crude product was purified by column chromatography on silica gel using hexane/EtOAc (1:1 v/v) to yield **6d** as a yellow solid (71.8 mg, 97%). mp 108–110 °C. IR (ATR): 3353, 1722, 1695, 1629 cm^–1^. ^1^H NMR (CDCl_3_, 400 MHz): δ 8.62 (dd, 1H, *J* = 1.2, 8.0 Hz), 8.41 (dd, 1H, *J* = 2.4, 8.8 Hz), 8.27 (d, 1H, *J* = 2.4 Hz), 7.66–7.57 (m, 3H), 7.52–7.43 (m, 2H), 3.67 (s, 3H), 2.41 (t, 2H, *J* = 7.2 Hz), 2.31 (t, 2H, *J* = 7.2 Hz), 1.79–1.71 (m, 2H), 1.66–1.59 (m, 2H), 1.44–1.29 (m, 8H). ^13^C{^1^H} NMR (CDCl_3_, 400 MHz): δ 179.9, 174.4, 172.0, 137.7, 137.3, 132.3, 132.1, 129.7, 129.3, 128.6, 126.9, 126.2, 126.1, 125.5, 119.1, 51.5, 37.7, 34.1, 29.2, 29.1, 29.0, 25.5, 24.9. (Several signals overlapped) HRMS (ESI-TOF) *m*/*z*: [M + Na]^+^ calcd for C_24_H_27_NNaO_4_S, 448.1559; found, 448.1557.

#### Synthesis of **6e**:

Compound **6e** was prepared from **6d** (58.3 mg, 0.137 mmol) and NaOH according to the procedure described for compound **6c**, and the crude product was purified by column chromatography on silica gel using hexane/EtOAc (1:2 v/v) to yield **6e** as a yellow solid (55.3 mg, 98%). mp 161–163 °C. IR (ATR): 3310, 1717, 1645, 1619 cm^–1^. ^1^H NMR (DMSO-*d*_6_, 400 MHz): δ 10.30 (br s, 1H), 8.72 (d, 1H, *J* = 2.4 Hz), 8.46 (dd, 1H, *J* = 1.2, 8.4 Hz), 8.04 (dd, 1H, *J* = 2.4, 8.4 Hz), 7.85–7.73 (m, 3H), 7.59–7.54 (m, 1H), 2.34 (t, 2H, *J* = 7.2 Hz), 2.15 (t, 2H, *J* = 7.2 Hz), 1.64–1.56 (m, 2H), 1.51–1.42 (m, 2H), 1.33–1.21 (m, 8H). ^13^C{^1^H} NMR (DMSO-*d*_6_, 100 MHz): δ 178.7, 174.6, 171.7, 138.3, 136.7, 132.9, 130.2, 129.1, 128.9, 128.0, 127.1, 126.64, 126.57, 124.6, 117.9, 36.4, 33.8, 28.7, 28.6, 25.0, 24.6. (Several signals overlapped) HRMS (ESI-TOF) *m*/*z*: [M + Na]^+^ calcd for C_23_H_25_NNaO_4_S, 434.1402; found, 434.1404.

### General Procedure for the Preparation of Compounds **7a**, **7b**, and **7c**

A mixture of 3-aminopropyl silica gel (1 g, 0.6–1.3 mmol, 1.0 equiv), **6a** (423 mg, 1.3 mmol, 1.0 equiv), 1*H*-benzotriazol-1-yloxytripyrrolidinophosphonium hexafluorophosphate (1.35 g, 2.6 mmol, 2.0 equiv), and *N,N*-diisopropylethylamine (1.1 mL, 6.5 mmol, 5.0 equiv) in DMF (13 mL, 0.1 M) was stirred at 23 °C for 15 h. After this time, the reaction was filtered, and the filtrate was redissolved in a solution of pyridine/acetic anhydride = 9:1 (13 mL, 0.1 M). After stirring at 23 °C for 16 h, the reaction was filtered, and the filtrate was concentrated in vacuo to yield **7a**.

**7b** was prepared according to the procedure described above for compound **7a.** For this purpose, **6c** (120 mg, 0.325 mmol) and 3-aminopropyl silica gel (250 mg) were employed.

**7c** was prepared according to the procedure described above for compound **7a.** For this purpose, **6e** (134 mg, 0.325 mmol) and 3-aminopropyl silica gel (250 mg) were employed.

### Evaluation of Compounds **D–F**

A solution containing *E*-**1a** (3.0 mg, 0.02 mmol) and **D**/**E**/**F** (5 mol%) in CH_3_CN (2 mL) was stirred in a photoreactor equipped with blue LEDs (425 nm, 18 W; PhotoRedOx Box EvoluChem, HepatoChem, Beverly, MA, USA) at room temperature for 15 min. The extent of isomerization was determined via ^1^H NMR spectroscopy performed at 296 K and 400 MHz.

### Isolation of the Diastereomers

Isolation of the various diastereomers was carried out using the HPLC conditions described as follows.

**1a**: YMC-Pack SIL-06, S-5 μm, 6 nm (φ 4.6 mm × 250 mm), eluent: CH_3_CN, flow rate: 1.0 mL/min, temperature: 23 °C, detection: 254 nm, initial peak retention time = 8.4 min for *Z*-**1a**, latter peak retention time = 11.5 min for *E*-**1a**.

**1b**: YMC-Pack SIL-06, S-5 μm, 6 nm (φ 4.6 mm × 250 mm), eluent: CH_3_CN, flow rate: 0.5 mL/min, temperature: 23 °C, detection: 254 nm, initial peak retention time = 12.7 min for *Z*-**1b**, latter peak retention time = 15.0 min for *E*-**1b**.

**1c**: YMC-Pack SIL-06, S-5 μm, 6 nm (φ 4.6 mm × 250 mm), eluent: CH_3_CN, flow rate: 0.5 mL/min, temperature: 23 °C, detection: 254 nm, initial peak retention time = 12.1 min for *Z*-**1c**, latter peak retention time = 14.3 min for *E*-**1c**.

### General Procedure for the Diastereomeric Enrichment of **1a–1c** Using the Recycling Photoreactor

*E*-**1a** (10 mg, 0.07 mmol) was injected into the recycling HPLC system equipped with a photoreactor. The fractions containing the desired diastereomer were accumulated over six cycles to yield *Z*-**1a** as a white solid (9.1 mg, 91%) with a *Z*/*E* ratio of 99:1.

*Z*-**1b** was prepared according to the procedure described above for *Z*-**1a**. Using *E*-**1b** (4 mg, 0.02 mmol), *Z*-**1b** (2.8 mg, 70%) was obtained as a colorless oil after four cycles. The obtained product possessed a *Z*/*E* ratio of 98:2.

*Z*-**1b** was prepared according to the procedure described above for *Z*-**1a**. Using *E*-**1b** (10 mg, 0.05 mmol), *Z*-**1b** (6.4 mg, 64%) was obtained as a colorless oil after nine cycles. The obtained product possessed a *Z*/*E* ratio of >99:1.

*Z*-**1c** was prepared according to the procedure described above for *Z*-**1a**. Using *E*-**1c** (10 mg, 0.05 mmol), *Z*-**1c** (6.8 mg, 68%) was obtained as a colorless oil after ten cycles. The obtained product possessed a *Z*/*E* ratio of >99:1.

## Data Availability

The data from this study are available in the published article and its Supporting Information.

## Supporting Information Available

Additional experimental details, materials, and methods: catalytic activities of compounds **D–F**; account of the photoreaction evaluation device; pseudo-first-order rate constants, *k*_obs_; UV–vis absorption spectra for compounds **1**–**3**; examination of the leaching of **7a**; account of the recycling photoreactor system; equilibrium states of **1a**, **1b**, and **1c**; enantiomeric enrichment of *Z*-**1a**, *Z*-**1b**, and *Z*-**1c**; and ^1^H, ^13^C, and 2D NMR (COSY, HMQC) spectra (PDF)
