# Supplementary material for: Isomerization of E-Cinnamamides into Z-Cinnamamides Using a Recycling Photoreactor
Source: J Org Chem. 2024 Jun 5;89(12):8836–44. doi: 10.1021/acs.joc.4c00721 (PMC11197087; doi:10.1021/acs.joc.4c00721)

Supporting Information

Isomerization of *E*-cinnamamides into *Z*-cinnamamides using a recycling photoreactor.

Mayuko Suga,[1] Saki Fukushima,[1] Kosho Makino,[2] Kayo Nakamura,[1] Hidetsugu Tabata,[3] Tetsuta Oshitari,[3] Hideaki Natsugari,[4] Noritaka Kuroda,[5] Kunio Kanemaru,[6] Yuji Oda,[6] and Hideyo Takahashi*[1]

[1]Faculty of Pharmaceutical Sciences, Tokyo University of Science, 2641 Yamazaki, Noda-shi, Chiba 278-8510, Japan

[2]Research Institute of Pharmaceutical Sciences, Musashino University, Nishitokyo, Tokyo 202-8585, Japan

[3]Faculty of Pharma Sciences, Teikyo University, 2-11-1 Kaga, Itabashi-ku, Tokyo 173-8605, Japan

[4]Graduate School of Pharmaceutical Science, The University of Tokyo, 7-3-1 Hongo, Bunkyo-ku, Tokyo 113-0033, Japan

[5]YMC Co., Ltd., 284 Daigo, Karasuma Nishiiru Gojo-dori, Shimogyo-ku, Kyoto 600-8106, Japan

[6]IWASAKI Electric Co., Ltd., 1-1, Ichiriyama-cho, Gyoda-shi, Saitama, 361-8505, Japan

*Corresponding Author: hide-tak@rs.tus.ac.jp

Contents

1. **Catalytic activities of compounds D, E, and F……….……….………………..S4**
2. **The photoreaction evaluation device………………………..………..... ......S4**
3. **Determination of the pseudo-first order rate constants (k_{obs}) of the isomerization of *E*-1a to *Z*-1a to evaluate the photosensitizers (thioxanthone, compounds 2–5) ………………………………………………………………....S5**
4. **UV-vis absorption spectra for thioxanthone and compounds 2–3……………………………………………………………………….…….....S8**
5. **Determination of the pseudo-first order rate constants (k_{obs}) of the isomerization of *E*-1a to *Z*-1a to evaluate the immobilized photosensitizers (compounds 7a, 7c, and 7e) ………………………………………………...…S9**
6. **Leaching of thioxanthone from immobilized catalyst 7a……………………………………………………………...………S10**
7. **Recycle of catalysts 7a………………………………………………………………S14**
8. **The recycling photoreactor system……………………………………………S15**
9. ***Z*/*E* ratio of cinnamamide 1a at equilibrium……………………..………...S15**
10. ***Z*/*E* ratio of Weinreb amide 1b at equilibrium…………………..……..….S16**
11. ***Z*/*E* ratio of Weinreb amide 1c at equilibrium……………………..……….S16**
12. **Isomeric enrichment of the alkene in the recycling photoreactor (Table 3)………………………………………………………………..…………S19**
13. **^{1}H, ^{13}C, and 2D NMR spectra of compounds *E*-1a, *E*-1b, *E*-1c, *Z*-1a, *Z*-1b, *Z*-1c, 4, 6a, 6b, 6c, 6d, 6e………………………………..…………………..…S23**

1. Catalytic activities of compounds D, E, and F

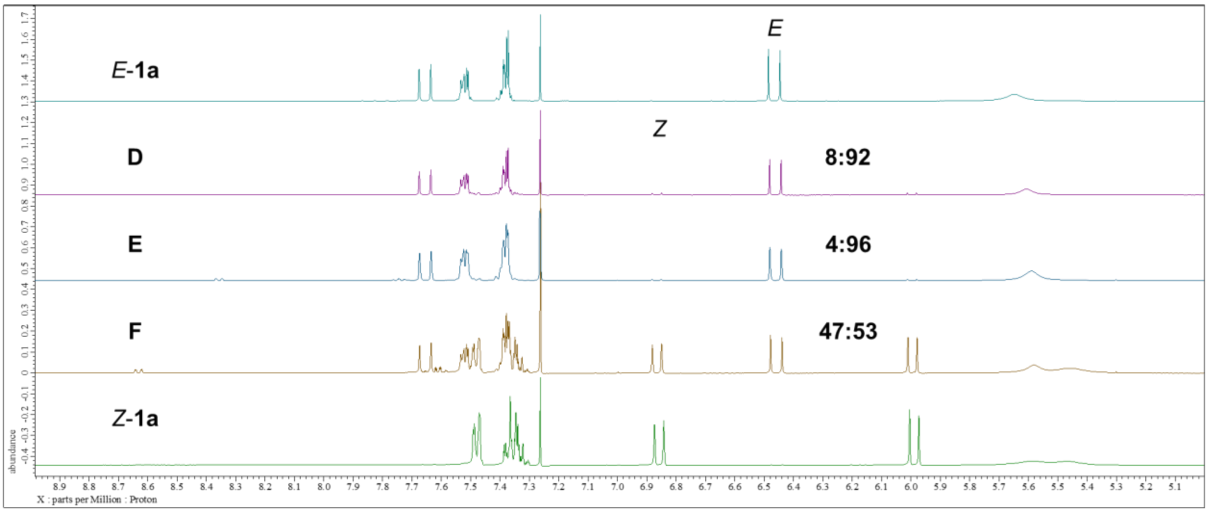

Figure S1. In the presence of 5 mol% catalyst (**D**, **E**, and **F**), a MeCN solution of *E*-**1a** was irradiated at 365 nm for 15 min. The *Z*/*E*-**1a** ratios after 15 min are shown.

2. The photoreaction evaluation device

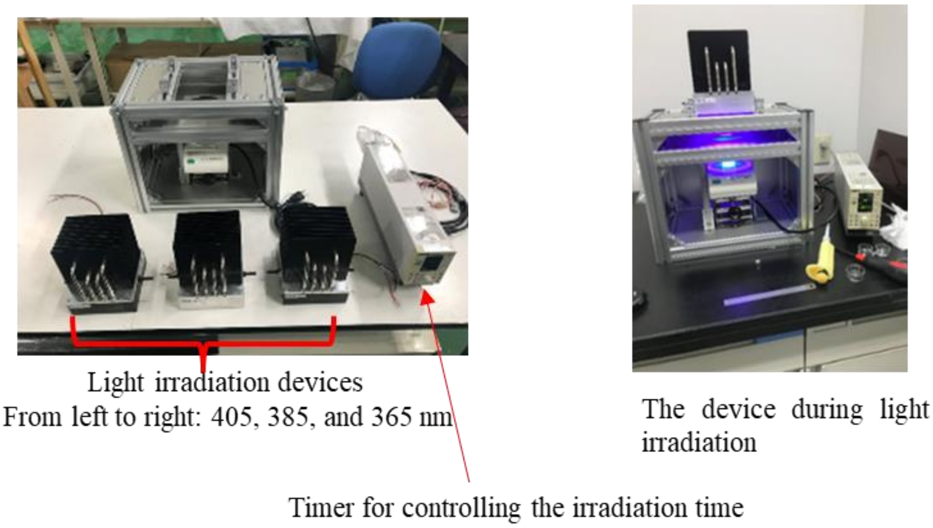

Figure S2. Photoreaction evaluation device with a controllable wavelength, illumination

intensity, and irradiation time.

3. Determination of the pseudo-first order rate constants (k_{obs}) of the isomerization of *E*-1a to *Z*-1a to evaluate the photosensitizers (thioxanthone, compounds 2–5)

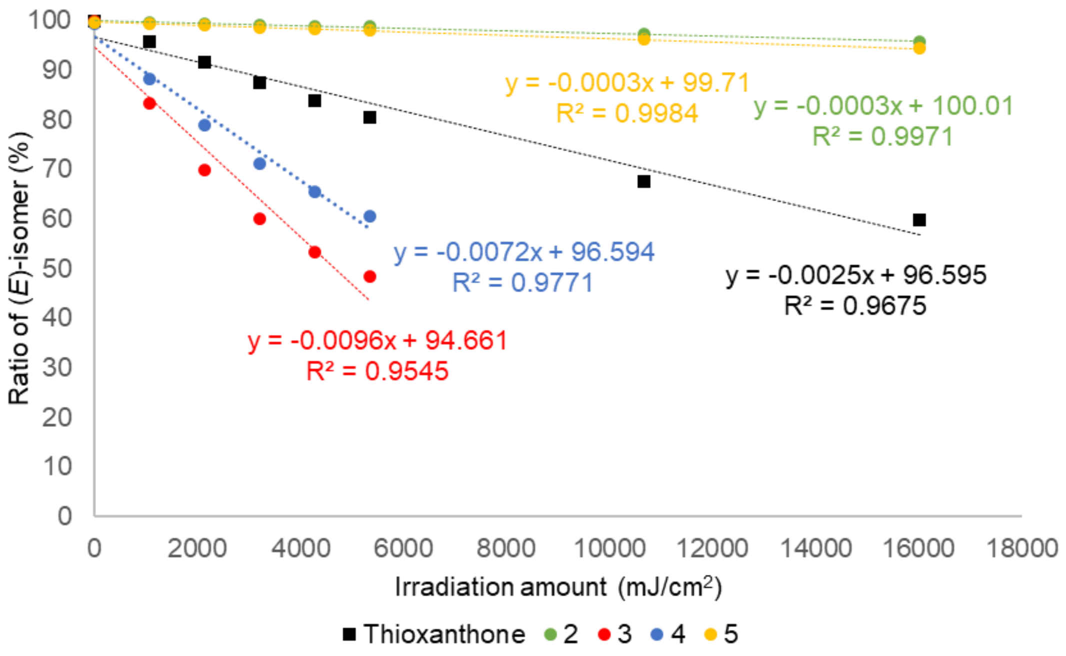

Figure S3. Isomerization rates (k_{obs}) of *E*-**1a** (0.01 M) in the presence of the various catalysts (thioxanthone, **2**, **3**, **4**, and **5**) at 23 °C in MeCN.

Time (min)	0	1	2	3	4	5	10	15
**Thioxanthone**	100	96	92	87	84	81	68	60
**2**	100	100	99	99	99	99	97	96
**3**	100	83	70	60	53	48	-	-
**4**	100	88	79	71	65	61	-	-
**5**	100	99	99	99	98	98	96	94

Table S1. Variation in the *E*-**1a** (%) over time in the presence of the various catalysts

The isomerization rates were shown to follow a pseudo-first order trend, and the rate constants, k_{obs}, were determined from the slope of the straight line obtained for a plot of $-\ln(\%ee_t/\%ee_0)$ against time.

$$y = ax + b$$

$$x = \frac{b - y}{-a}$$

$$y = 55$$

$$x = \frac{b - 55}{-a}$$

Using the above formulae, the integrated irradiance of compound **2** (Table 2, entry 2) was calculated as follows:

$$y = -0.0003x + 100.01$$

$$x = \frac{100.01 - y}{0.0003}$$

$$y = 55$$

$$x = \frac{100.01 - 55}{0.0003}$$

$$x \fallingdotseq 1.50 \times 10^5$$

The integrated irradiances of all other compounds were calculated in a similar manner.

Compounds *E*-**1a** and *Z*-**1a** were separated using chiral HPLC (YMC-Pack SIL-06, S-5 µm, 6 nm; φ4.6 mm × 250 mm) with MeCN as the eluent, and a flow rate of 1.0 mL/min. The retention times (*t*R) of *E*-**1a** and *Z*-**1a** were 11.5 and 8.35 min, respectively.

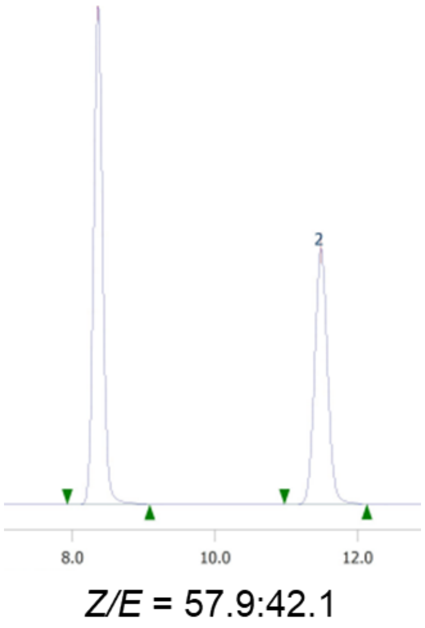

Figure S4. Chromatogram for the HPLC separation of *E*-**1a** and *Z*-**1a.**

4. UV-vis absorption spectra for thioxanthone and compounds 2–3

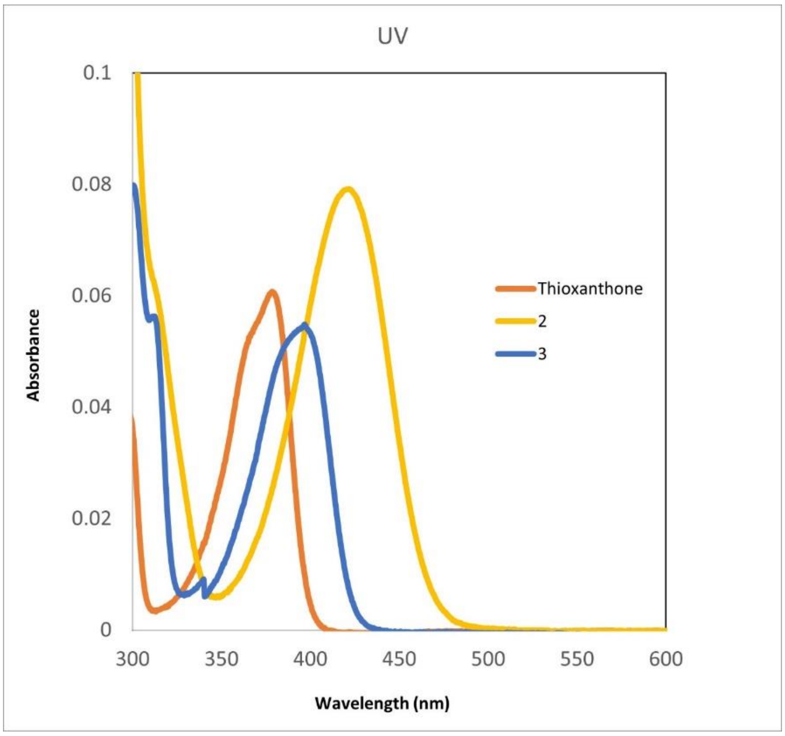

Figure S5. UV-vis absorption spectra for thioxanthone, and compounds **2–3**.

5. Determination of the pseudo-first order rate constants (kobs) of the isomerization of E-1a to Z-1a to evaluate the immobilized photosensitizers (compounds 7a, 7c, and 7e)

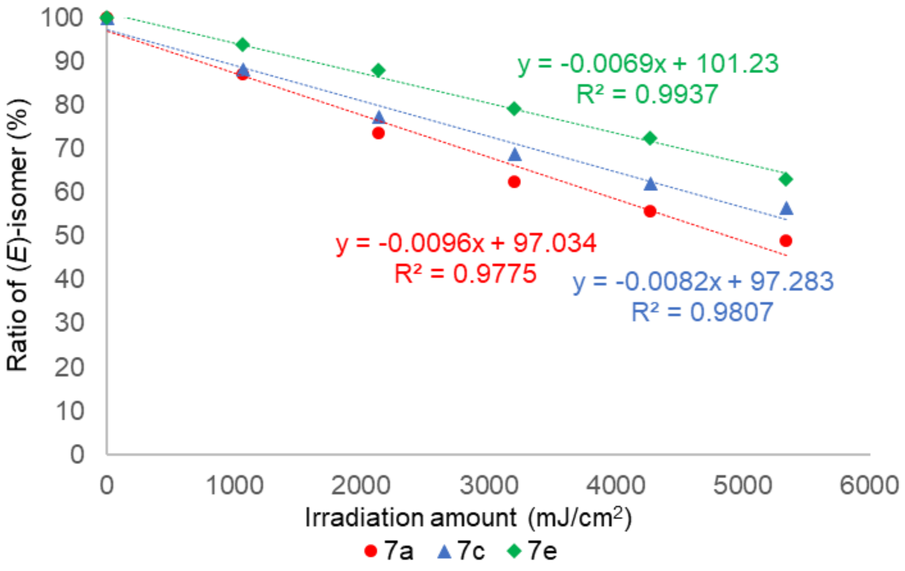

Figure S6. Isomerization rates (k_{obs}) of *E*-**1a** (0.01 M) in the presence of catalysts **7a**, **7c**, and **7e** at 23 °C in MeCN.

Table S2. Variation in the *E*-**1a** (%) over time in the presence of catalysts **7a–7c**

Time (min)	0	1	2	3	4	5
**7a**	100	94	88	79	72	63
**7c**	100	88	77	69	62	56
**7e**	100	87	74	62	56	49

$$y = ax + b$$

$$x = \frac{b - y}{-a}$$

$$y = 55$$

$$x = \frac{b - 55}{-a}$$

Using the above formulae, the integrated irradiance of compound **7a** (Table 2, entry 6) was calculated as follows:

$$y = -0.0096x + 97.034$$

$$x = \frac{97.034 - y}{0.0096}$$

$$y = 55$$

$$x = \frac{97.034 - 55}{0.0096}$$

$$x \risingdotseq 4.38 \times 10^3$$

The integrated irradiances of all other compounds were calculated in a similar manner.

6. Leaching of thioxanthone from immobilized catalyst 7a

The leaching test of **7a** was carried out according to the following reaction and the corresponding chromatograms are presented below.

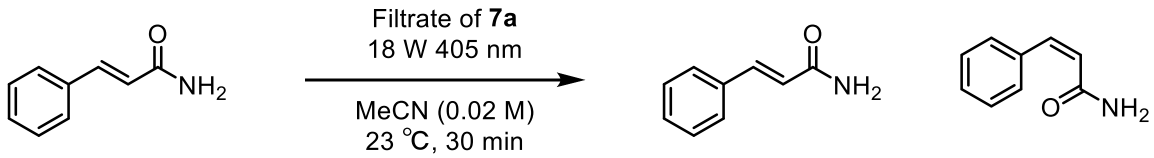

E-**1a** *E*-**1a** *Z*-**1a**

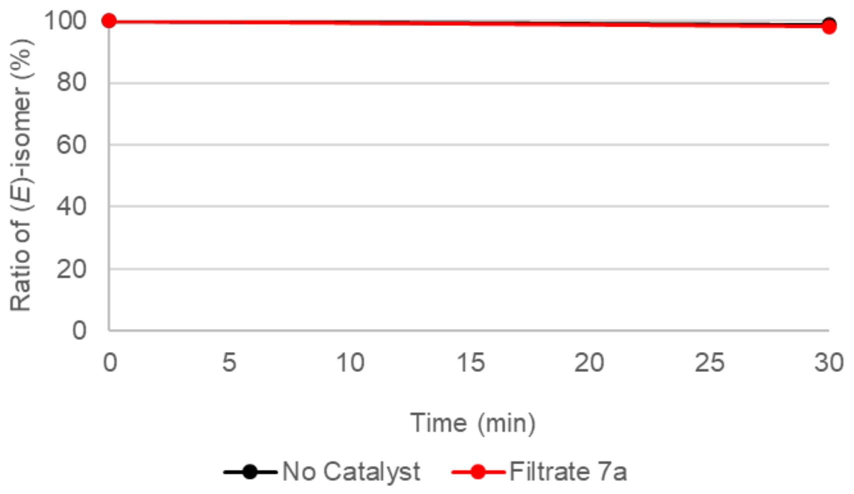

Figure S7. Variation in the *E*-**1a** ratio in the presence of the filtrate of **7a** and in the absence of catalyst.

Chromatogram 1: At 0 min in the absence of catalyst; YMC-Pack SIL-06, S-5 μm, 6 nm (φ4.6 mm × 250 mm); MeCN eluent; 1.0 mL/min flow rate;. *t*R: 13.2 min for *E*-**1a**, 9.78 min for *Z*-**1a**

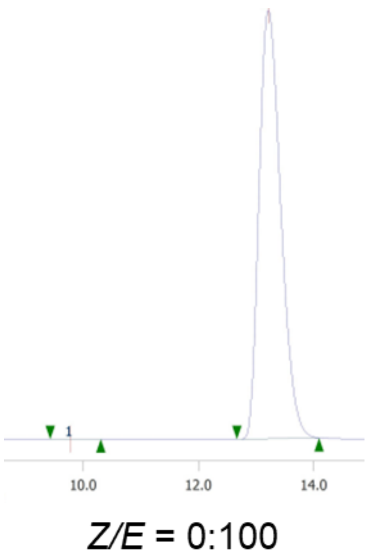

Chromatogram 2: At 30 min in the absence of catalyst; YMC-Pack SIL-06, S-5 µm, 6 nm (φ4.6 mm × 250 mm); MeCN eluent; 1.0 mL/min flow rate;. *t*R: 13.2 min for *E*-**1a**, 9.80 min for *Z*-**1a**

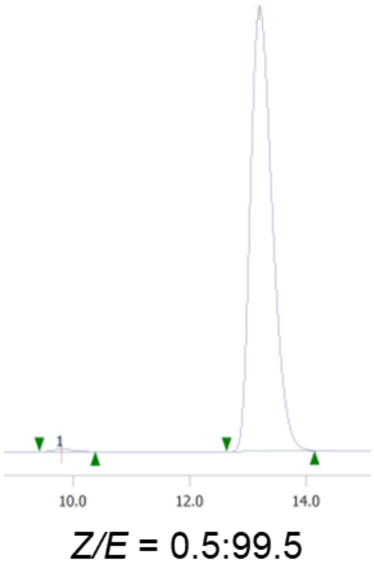

Chromatogram 3: At 0 min in the presence of **7a**; YMC-Pack SIL-06, S-5 µm, 6 nm (φ4.6 mm × 250 mm); MeCN eluent; 1.0 mL/min flow rate;. *t*R: 12.2 min for *E*-**1a**, 10.2 min for *Z*-**1a**

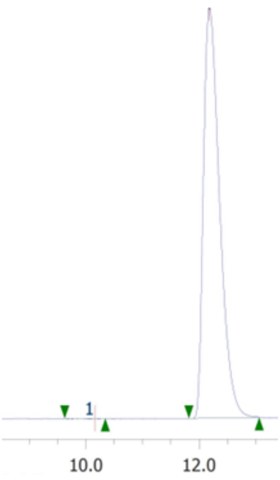

Z/E = 0:100

Chromatogram 4: At 30 min in the presence of **7a**; YMC-Pack SIL-06, S-5 µm, 6 nm (φ4.6 mm × 250 mm); MeCN eluent; 1.0 mL/min flow rate;. *t*R: 11.0 min for *E*-**1a**, 8.18 min for *Z*-**1a**

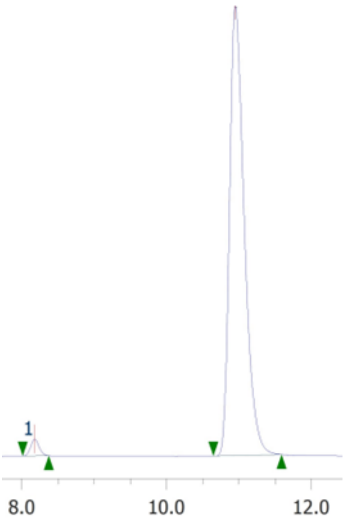

Z/E = 2.1:97.9

7. Recycle of catalysts 7a.

Table S3

Isomerization of **1a** by recycled immobilized catalyst **7a**

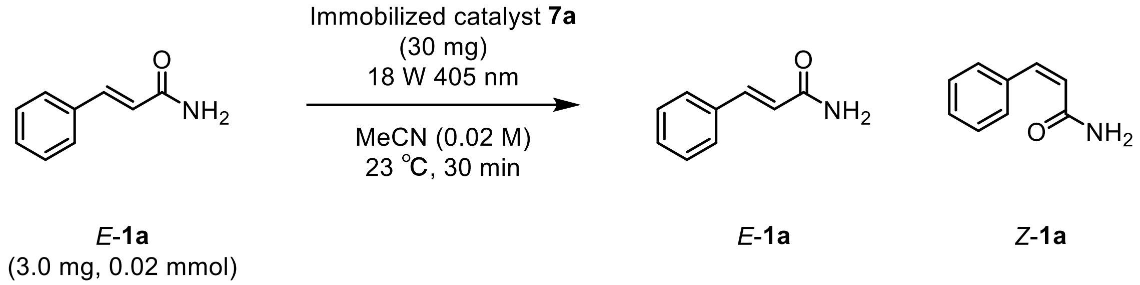

YMC-Pack SIL-06, S-5 µm, 6 nm (φ4.6 mm × 250 mm)

Eluent: MeCN Flow rate : 0.5 mL/min. *t*R: 16.2 min for *E*-**1a**, 19.0 min for *Z*-**1a**

Number of use	1	2	3	4	5	6	7	8	9	10
Z/E	46:54	45:55	51:49	47:53	40:60	41:59	47:53	47:53	51:49	51:49

8. The recycling photoreactor system

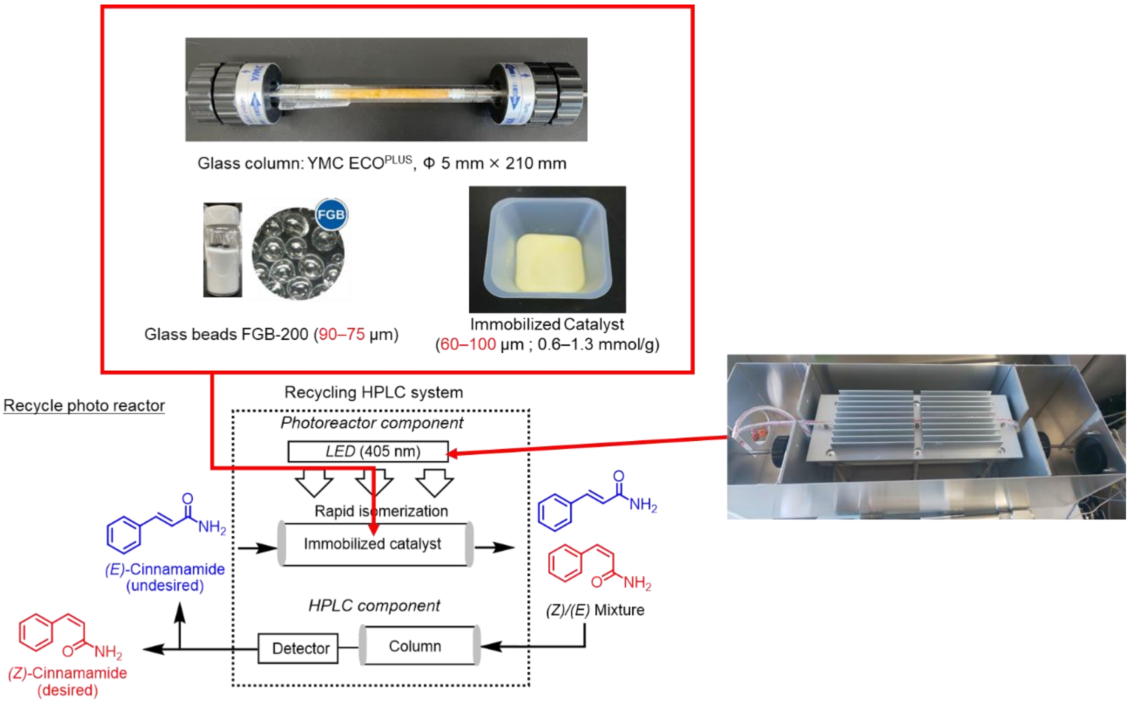

Figure S8. The recycling photoreactor system employed in the current study.

9. *Z/E* ratio of cinnamamide 1a at equilibrium

The reaction progress was monitored by HPLC during irradiation with LED light (405 nm) for 15 min, at which point the isomerization process reached an equilibrium state (*Z*/*E* = ~60/40).

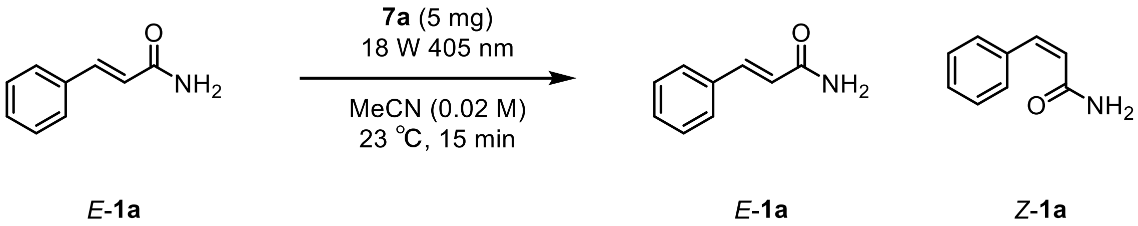

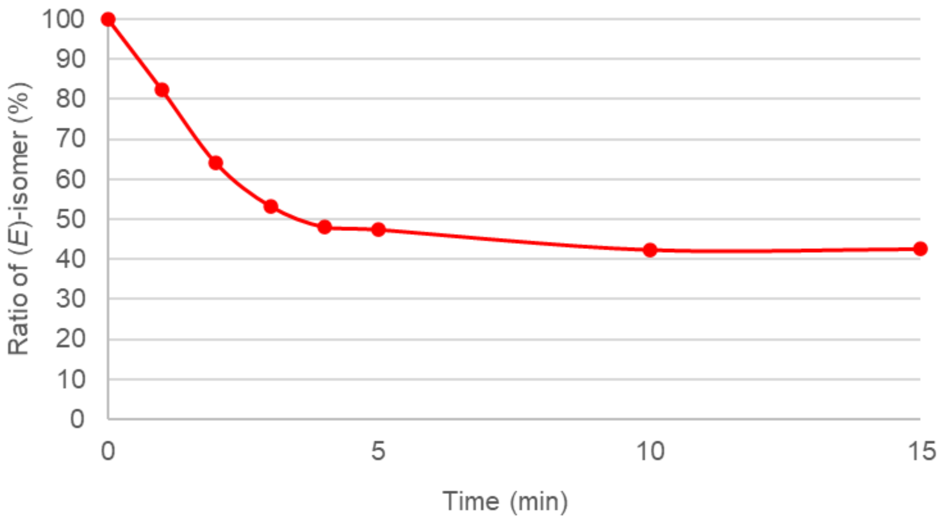

Figure S9. Variation in the ratio of *E*-**1a** under LED irradiation for 15 min.

10. *Z*/*E* ratio of Weinreb amide 1b at equilibrium

The reaction progress was monitored by HPLC during irradiation with LED light (405 nm) for 15 min, at which point the isomerization process reached an equilibrium state (*Z*/*E* = ~72/28).

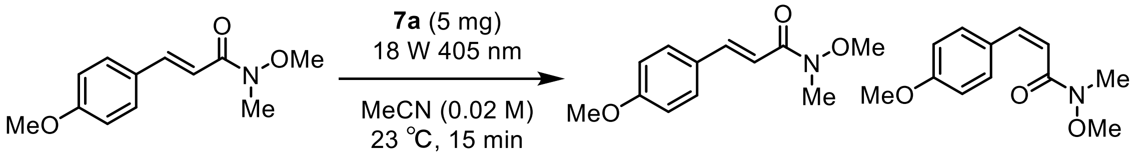

E-**1b** *E*-**1b** *Z*-**1b**

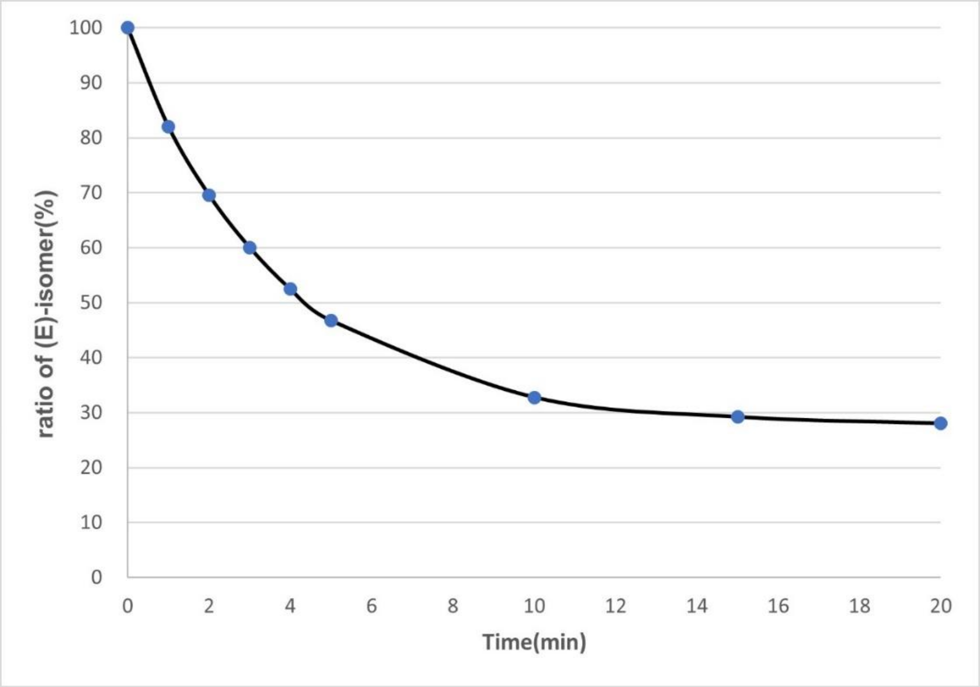

Figure S10. Variation in the ratio of *E*-**1b** under LED irradiation for 15 min.

11. *Z*/*E* ratio of Weinreb amide 1c at equilibrium

The reaction progress was monitored by HPLC during irradiation with LED light (405 nm) for 15 min, at which point the isomerization process reached an equilibrium state (*Z*/*E* = ~78/22).

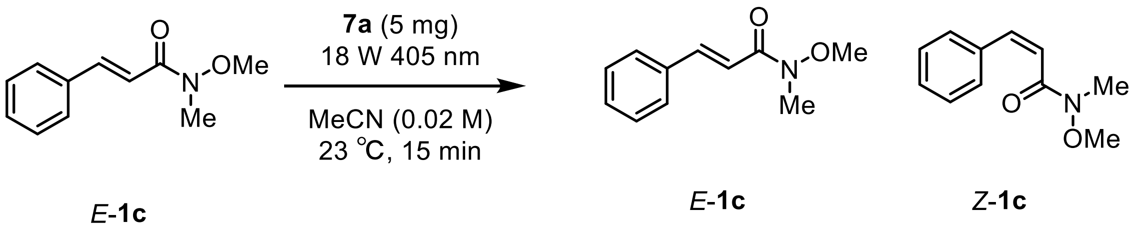

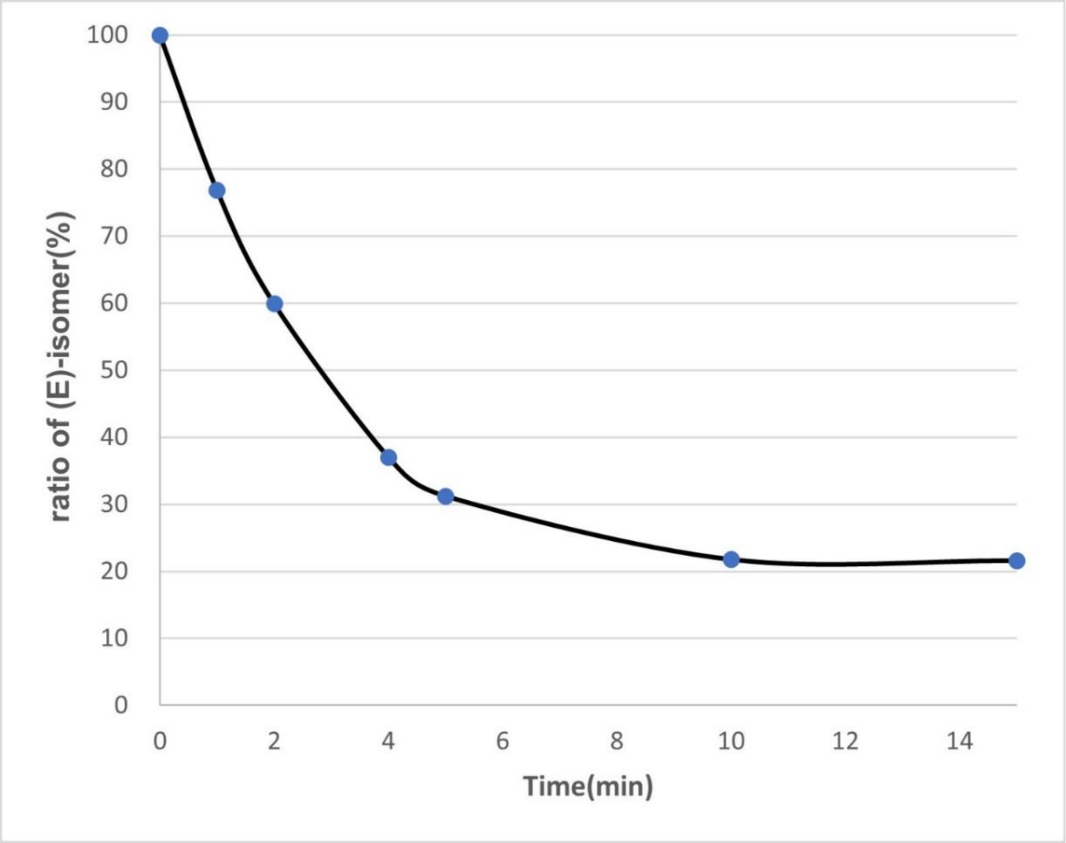

Figure S11. Variation in the ratio of *E*-**1c** under LED irradiation for 15 min.

12. Isomeric enrichment of the alkene in the recycling photoreactor (Table 3)

Entry 1 (1a)

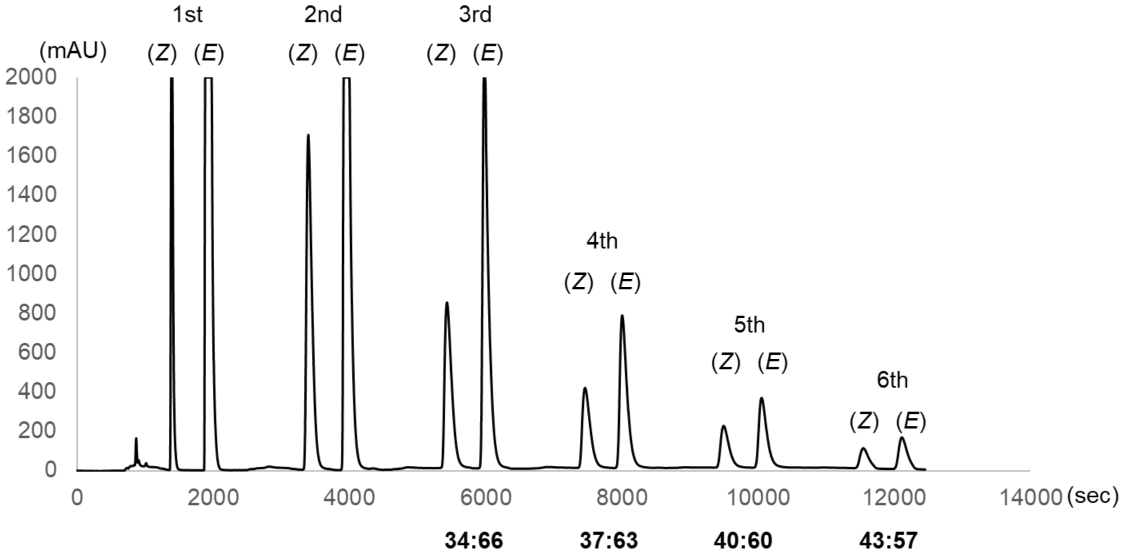

Figure S12. With accumulation of the fraction of the *Z*-isomer. Column: YMC-Pack SIL-06 (f 20 mm, length 25 cm), Eluent: CH_3CN, Flow rate: 4.7 mL/min.

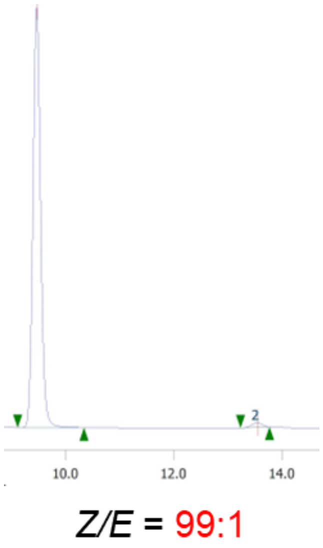

Figure S13. YMC-Pack SIL-06, S-5 µm, 6 nm (φ4.6 mm × 250 mm); MeCN eluent; 1.0 mL/min flow rate;. *t*R: 13.5 min for *E*-**1a**, 9.47 min for *Z*-**1a.**

Entry 2 (1b)

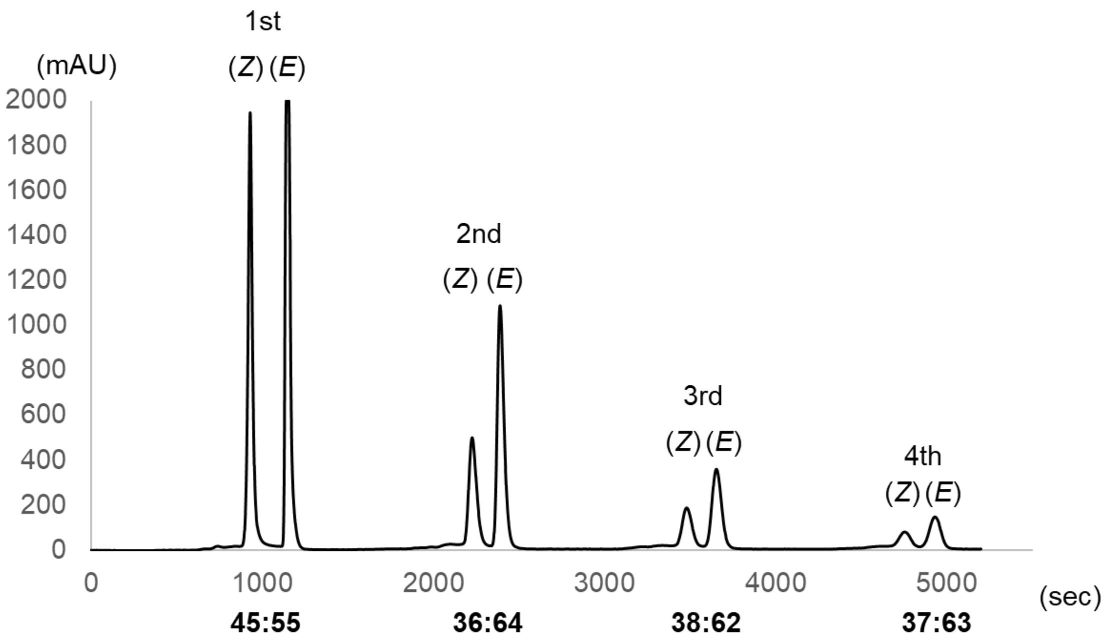

Figure S14. With accumulation of the fraction of the *Z*-isomer. Column: YMC-Pack SIL-06 (f 20 mm, length 25 cm), Eluent: CH_3CN, Flow rate: 4.7 mL/min.

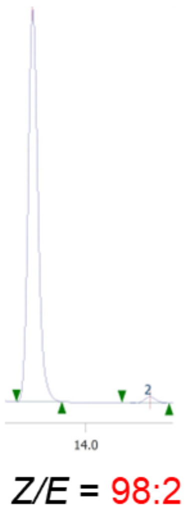

Figure S15. YMC-Pack SIL-06, S-5 µm, 6 nm (φ4.6 mm × 250 mm); MeCN eluent; 1.0 mL/min flow rate;. *t*R: 15.3 min for *E*-**1b**, 12.9 min for *Z*-**1b.**

Entry 3 (1b)

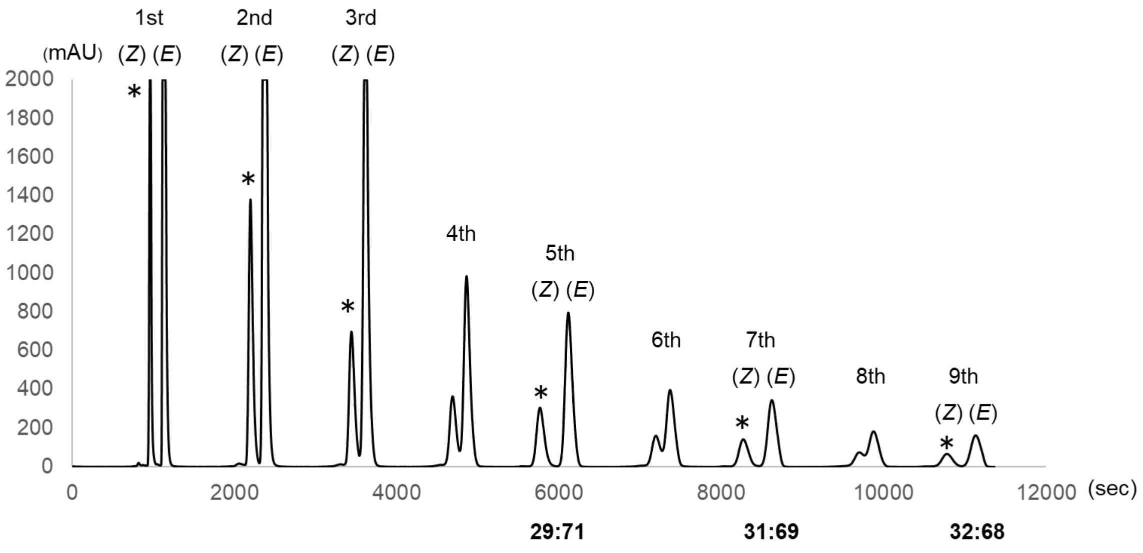

Figure S16. The fraction denoted by an asterisk is accumulated. Column: YMC-Pack SIL-06 (f 20 mm, length 25 cm), Eluent: CH_3CN, Flow rate: 4.7 mL/min.

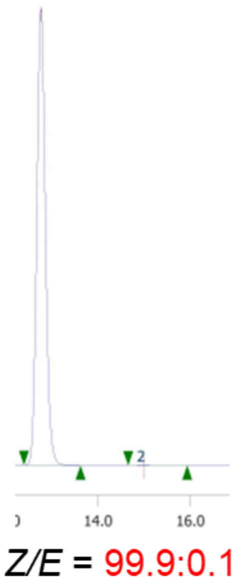

Figure S17. YMC-Pack SIL-06, S-5 μm, 6 nm (φ4.6 mm × 250 mm); MeCN eluent; 1.0 mL/min flow rate;. *t*R: 15.0 min for *E*-**1b**, 12.8 min for *Z*-**1b.**

Entry 4 (1c)

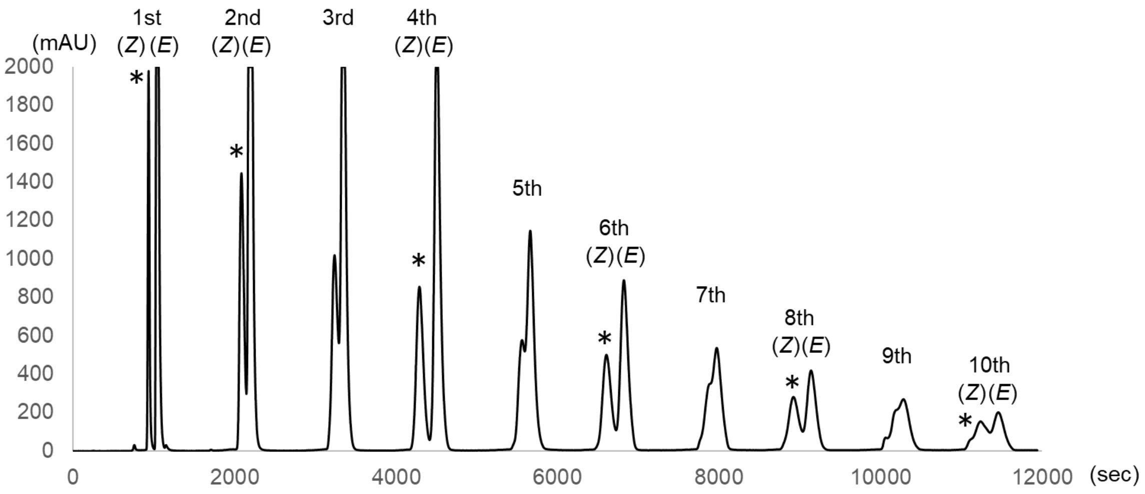

Figure S18. The fraction denoted by an asterisk is accumulated. Column: YMC-Pack SIL-06 (f 20 mm, length 25 cm), Eluent: CH_3CN, Flow rate: 4.7 mL/min.

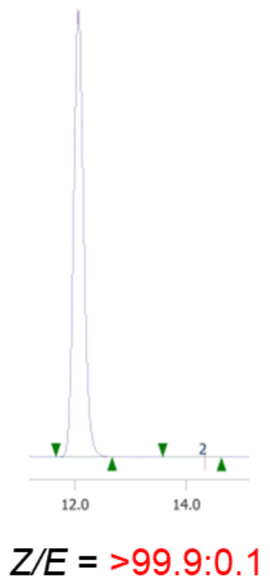

Figure S19. YMC-Pack SIL-06, S-5 μm, 6 nm (φ4.6 mm × 250 mm); MeCN eluent; 1.0 mL/min flow rate;. *t*R: 14.3 min for *E*-**1c**, 12.1 min for *Z*-**1c.**

13. ^{1}H, ^{13}C, and 2D NMR spectra of compounds *E*-1a, *E*-1b, *E*-1c, *Z*-1a, *Z*-1b, *Z*-1c, 4, 6a, 6b, 6c, 6d, 6e

^{1}H NMR (400 MHz, $CDCl_3$) ***E*-1a**

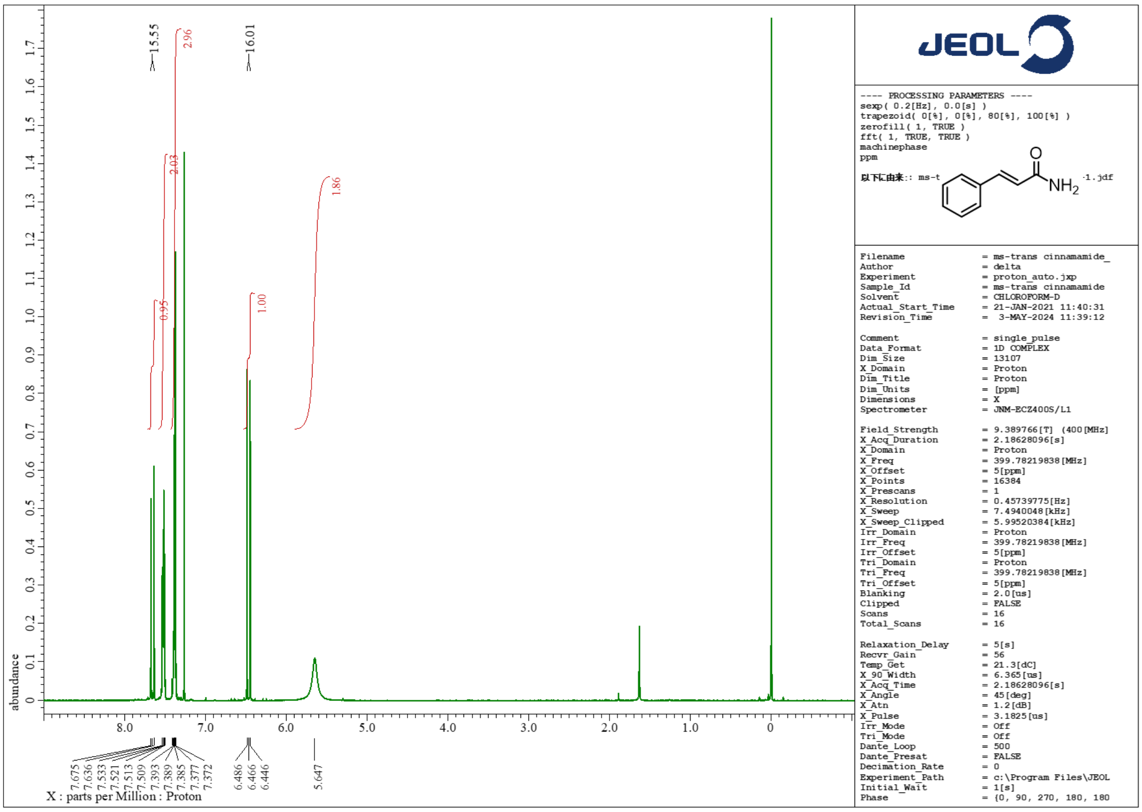

$^{13}C\{^1H\}$ NMR (150 MHz, $CDCl_3$) ***E*-1a**

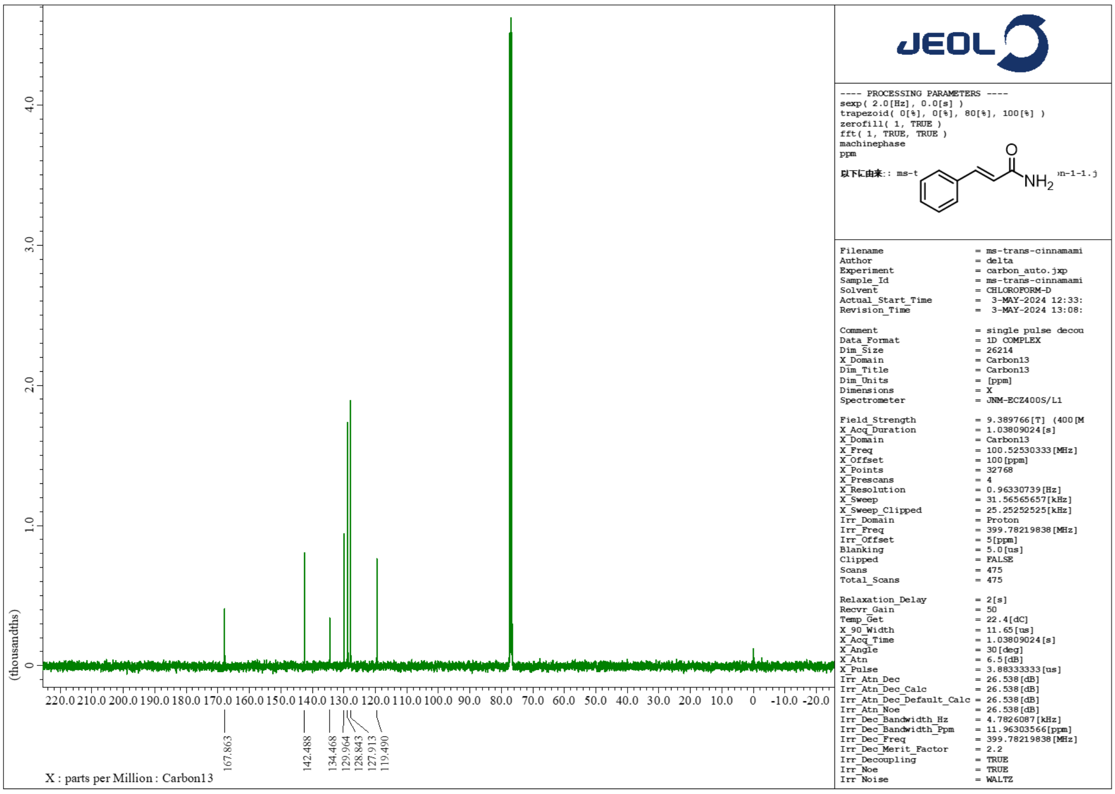

^{1}H NMR (400 MHz, $CDCl_3$) ***E*-1b**

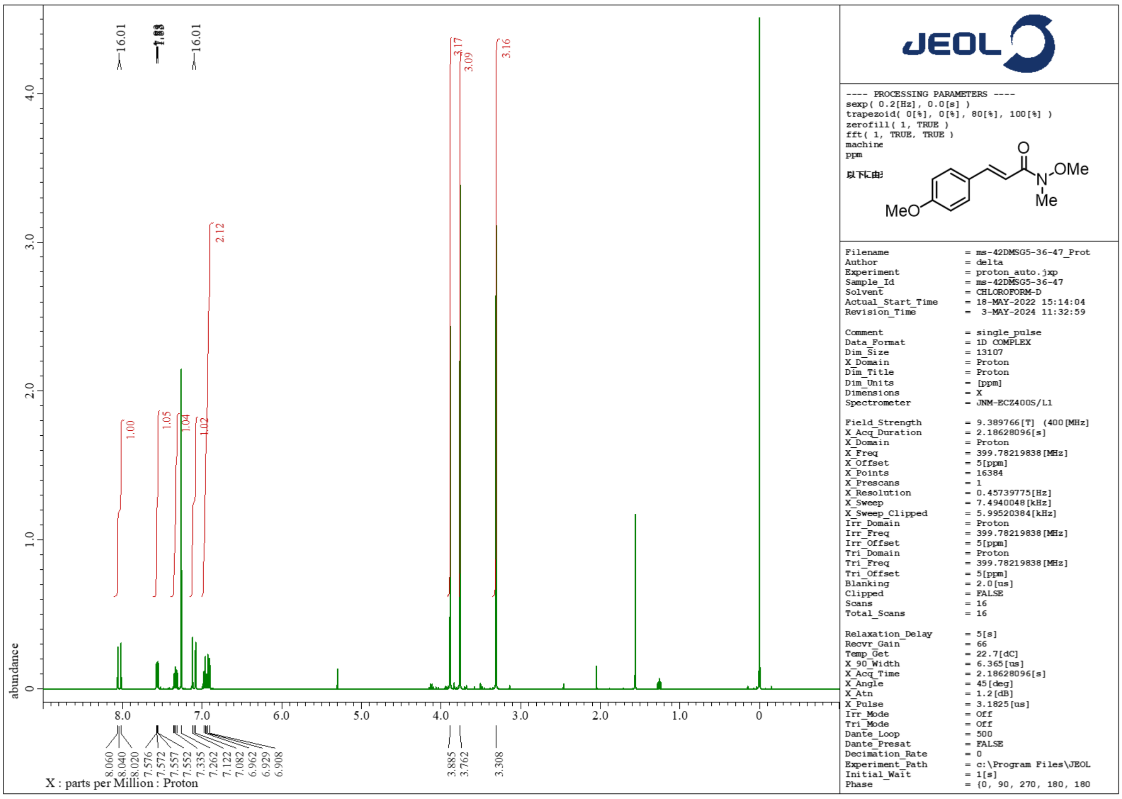

$^{13}C\{^{1}H\}$ NMR (150 MHz, $CDCl_3$) ***E*-1b**

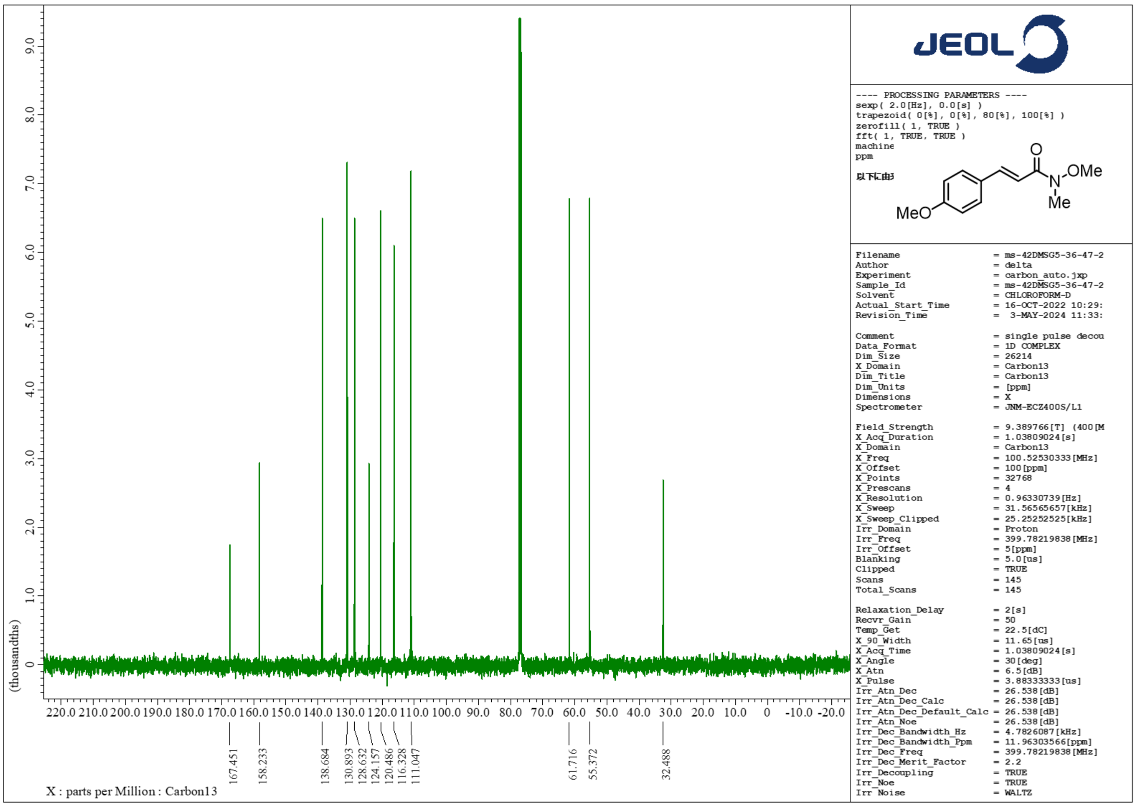

^{1}H NMR (400 MHz, $CDCl_3$) ***E*-1c**

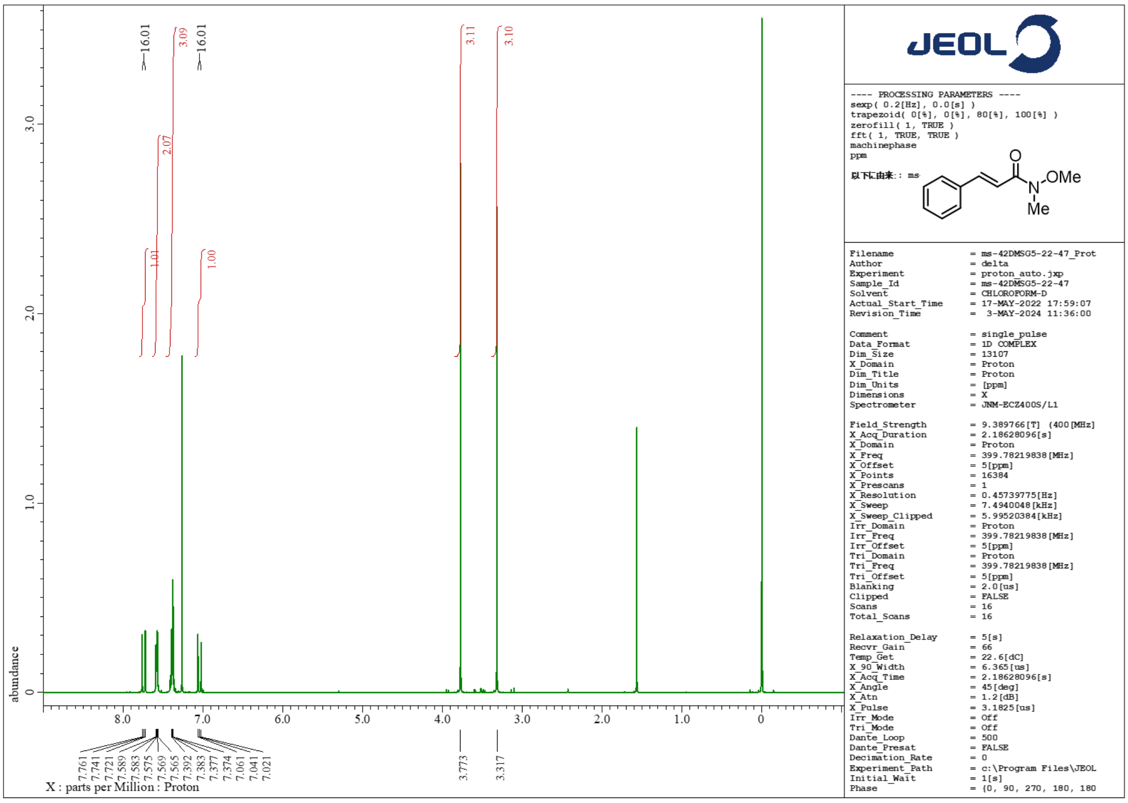

$^{13}C\{^{1}H\}$ NMR (150 MHz, $CDCl_3$) ***E*-1c**

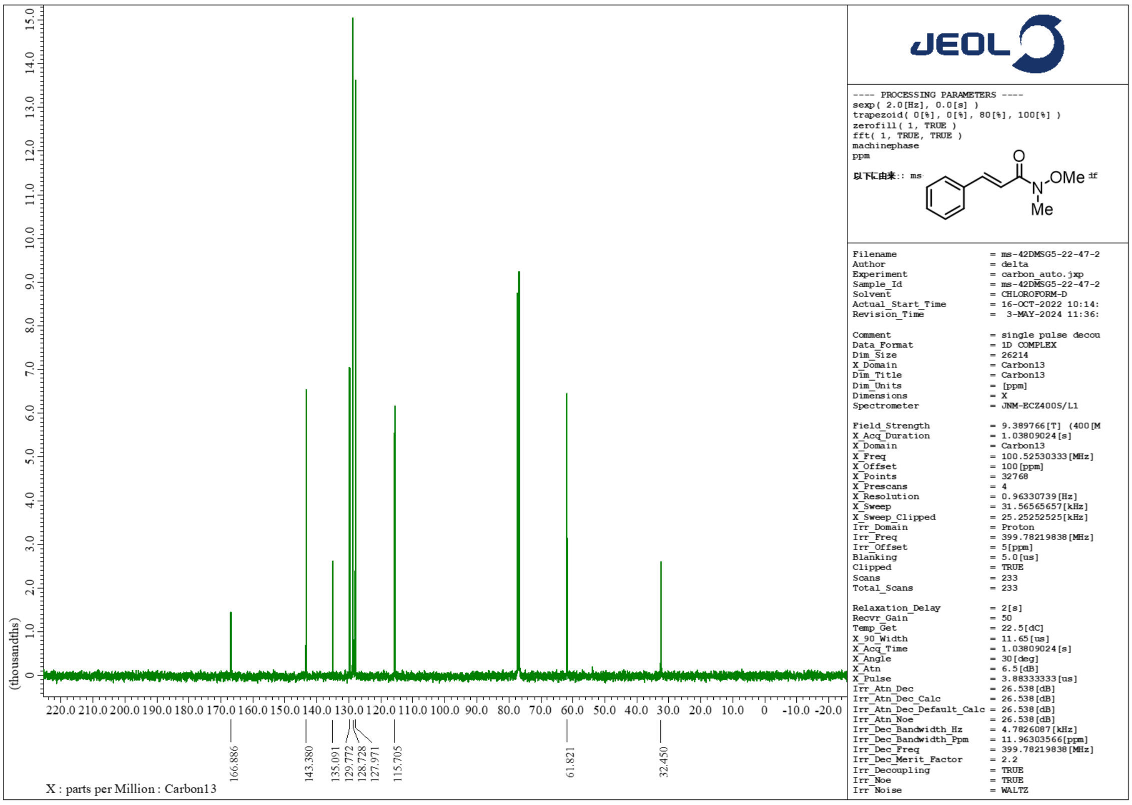

^{1}H NMR (400 MHz, $CDCl_3$) ***Z*-1a**

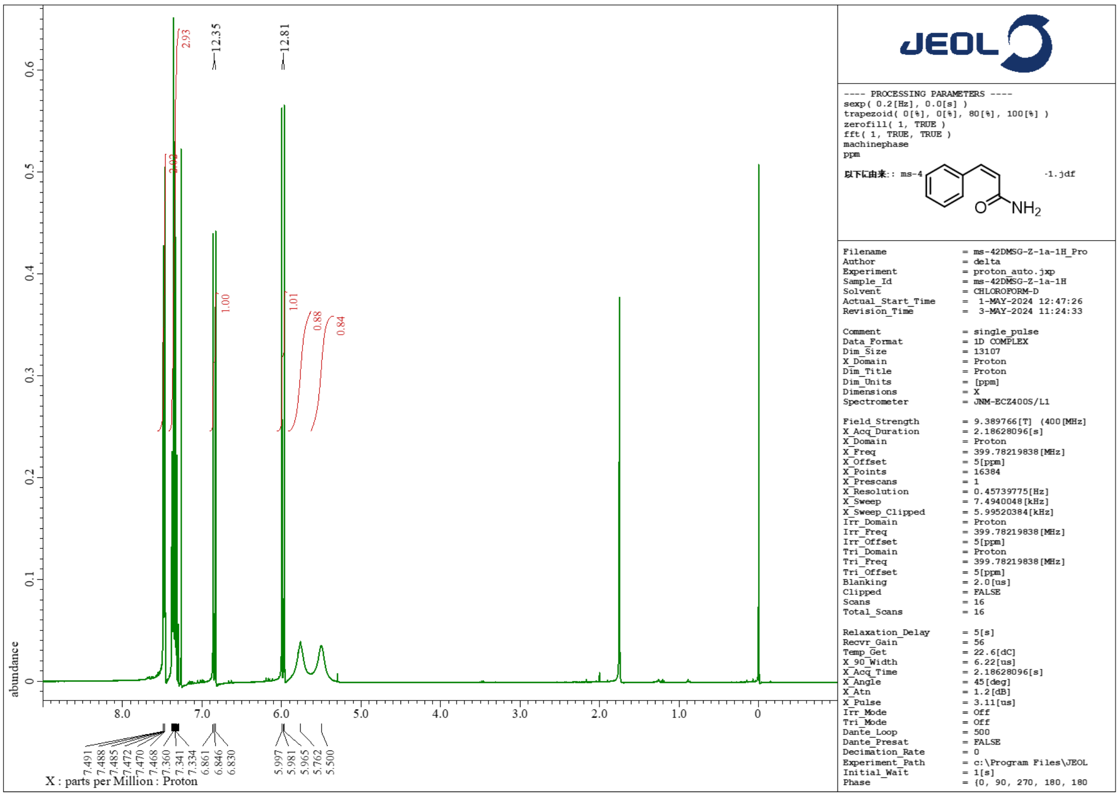

$^{13}C\{^{1}H\}$ NMR (150 MHz, $CDCl_3$) ***Z*-1a**

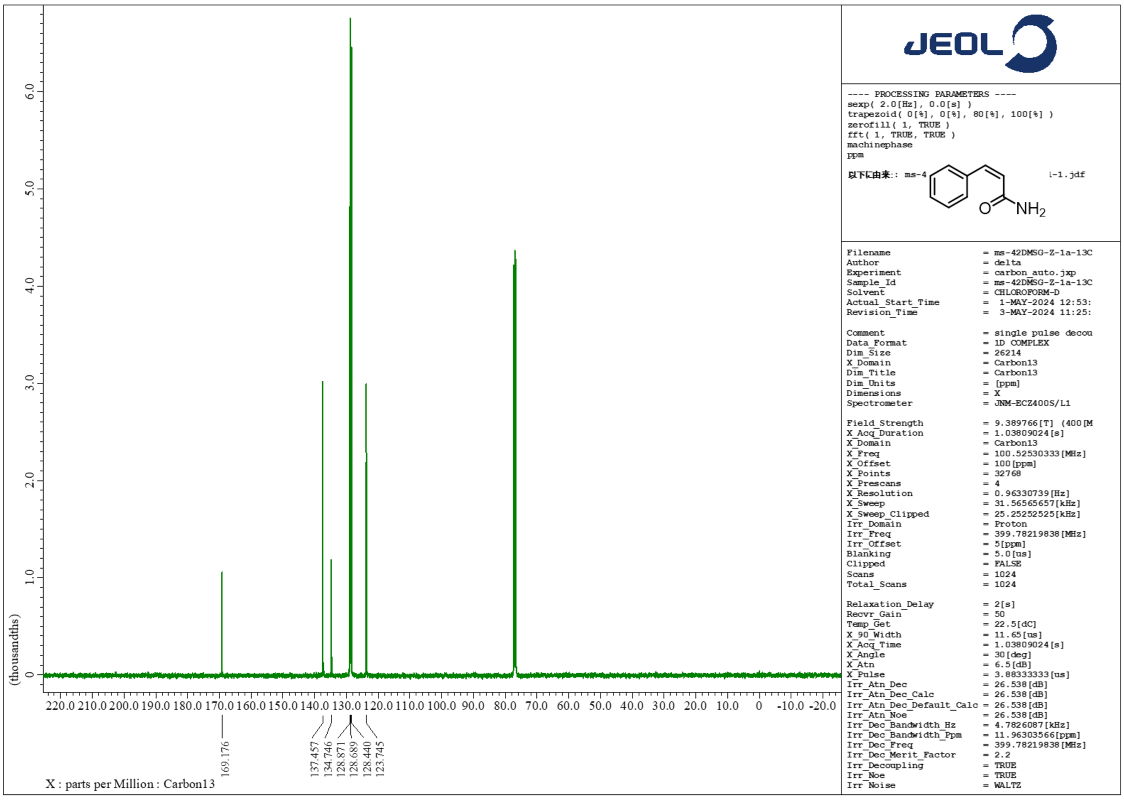

^{1}H NMR (600 MHz, $CDCl_3$) ***Z*-1b**

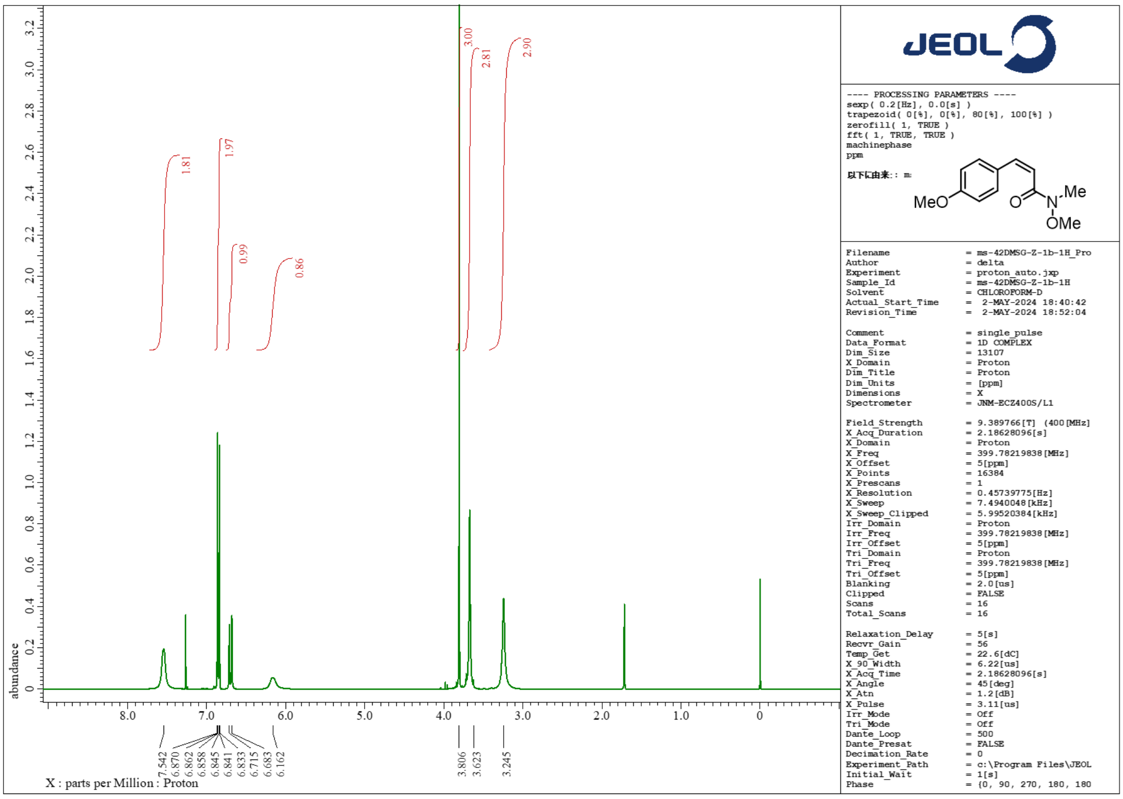

$^{13}C\{^{1}H\}$ NMR (150 MHz, $CDCl_3$) ***Z*-1b**

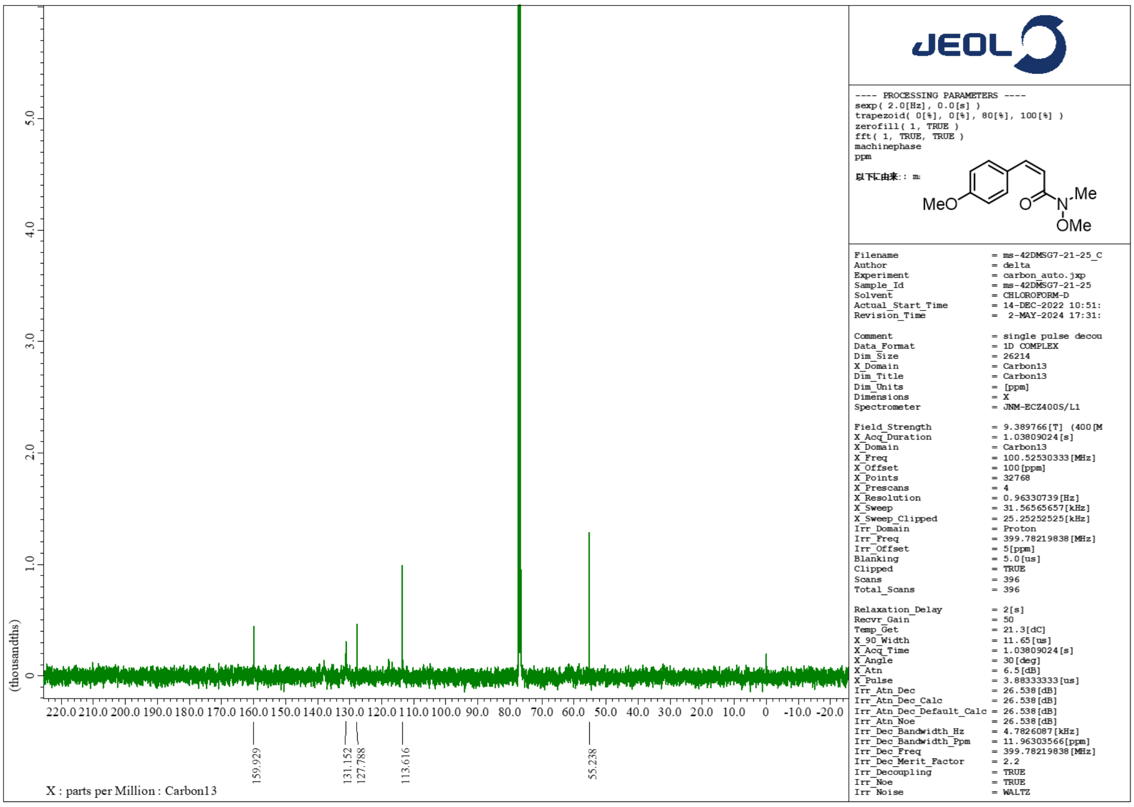

^{1}H-^{1}H COSY NMR (600 MHz, $CDCl_3$) ***Z*-1b**

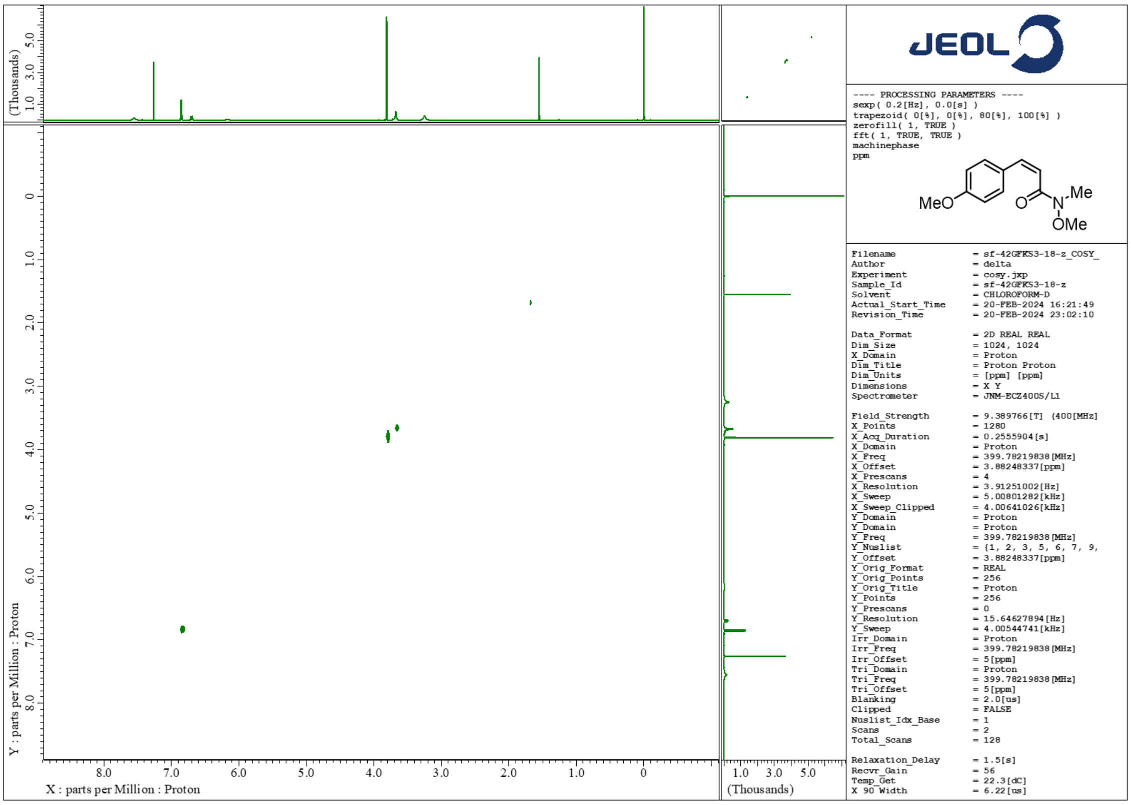

^{1}H-^{13}C HMQC NMR ($CDCl_3$) ***Z*-1b**

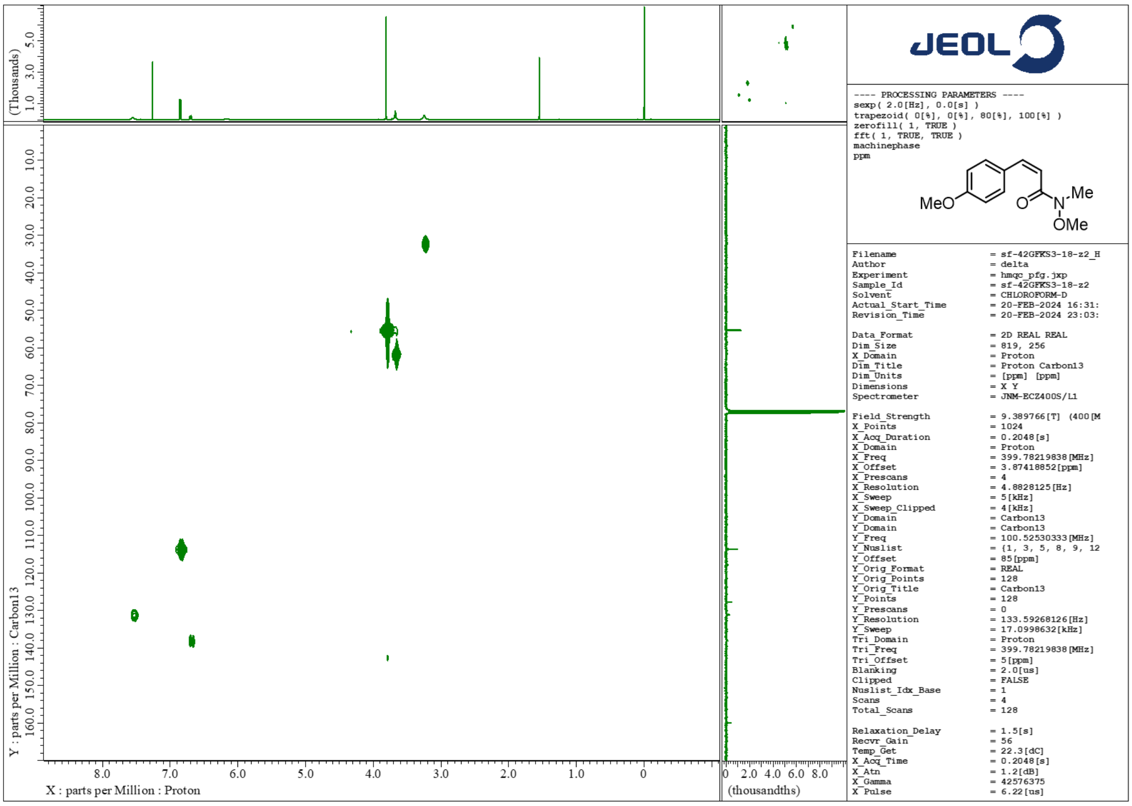

^{1}H NMR (400 MHz, $CDCl_3$) ***Z*-1c**

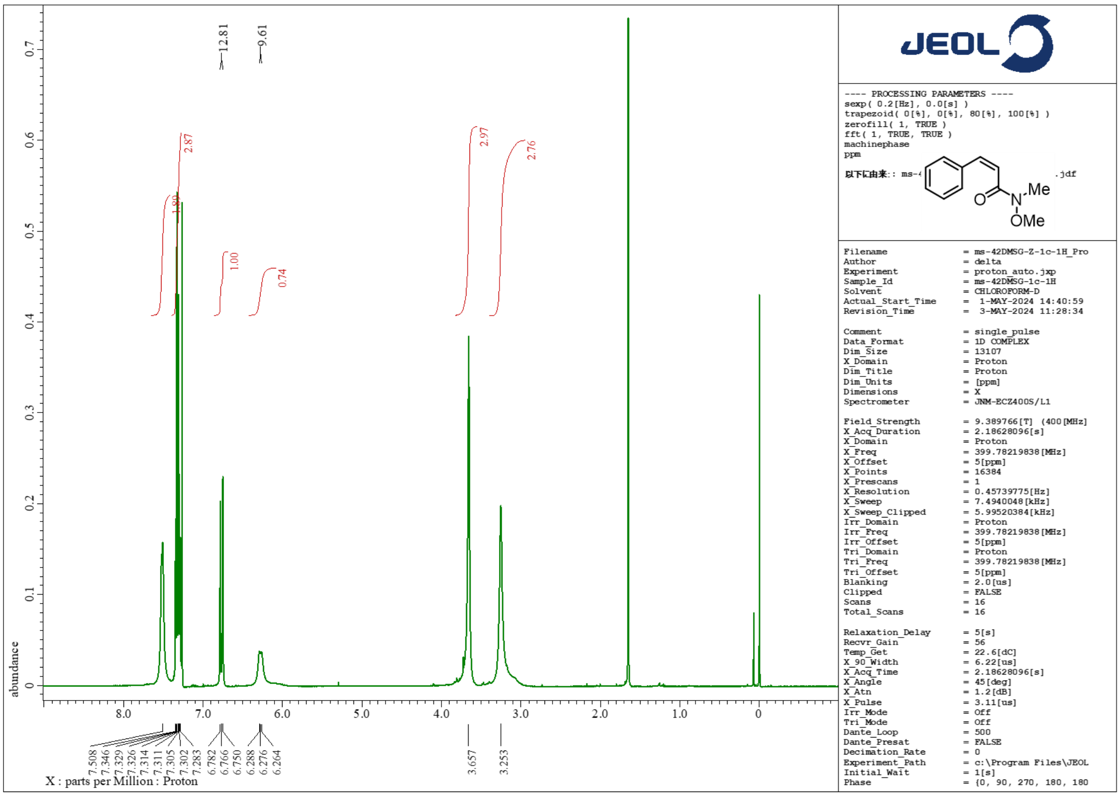

$^{13}C\{^{1}H\}$ NMR (150 MHz, $CDCl_3$) ***Z*-1c**

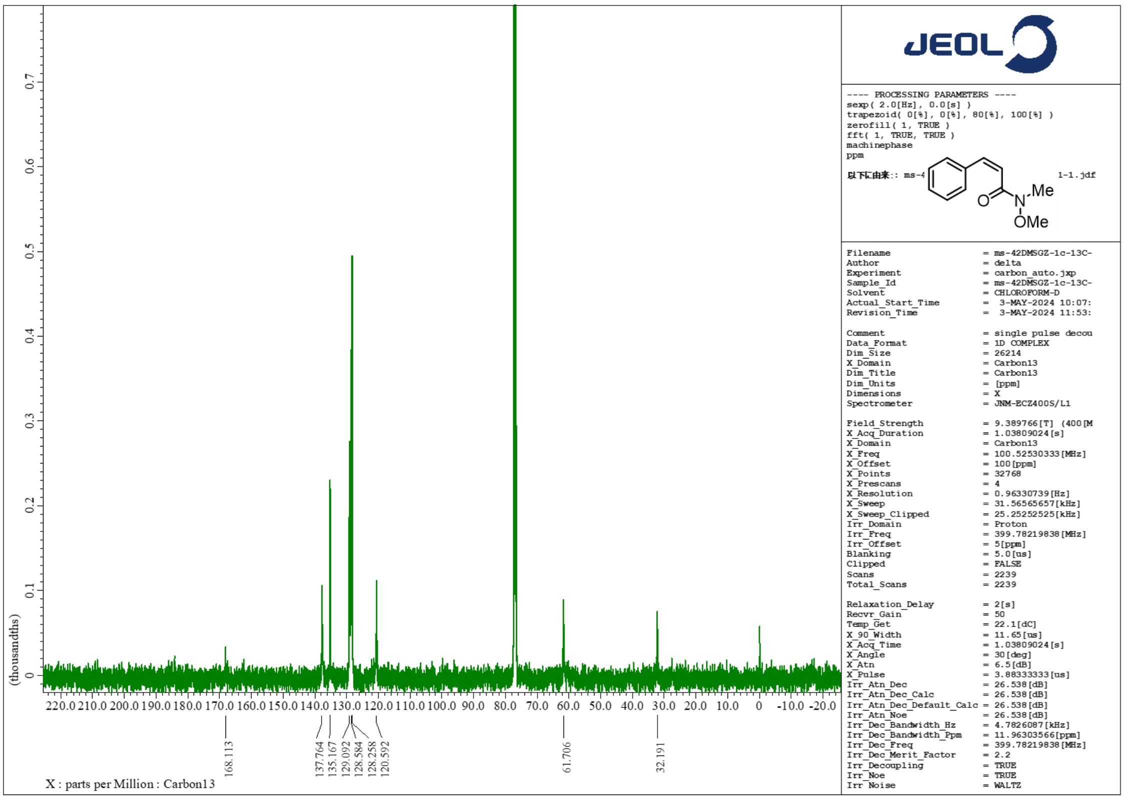

^{1}H NMR (400 MHz, DMSO-d_6) **4**

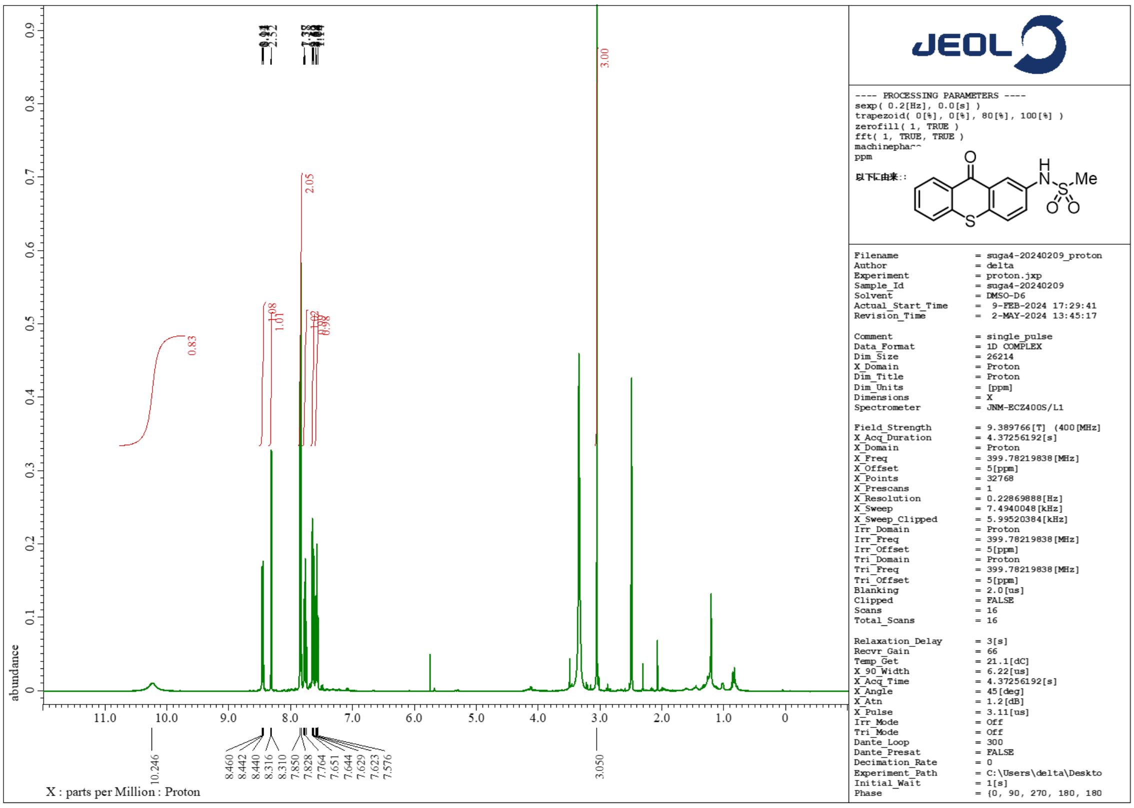

$^{13}C\{^1H\}$ NMR (150 MHz, DMSO-d_6) **4**

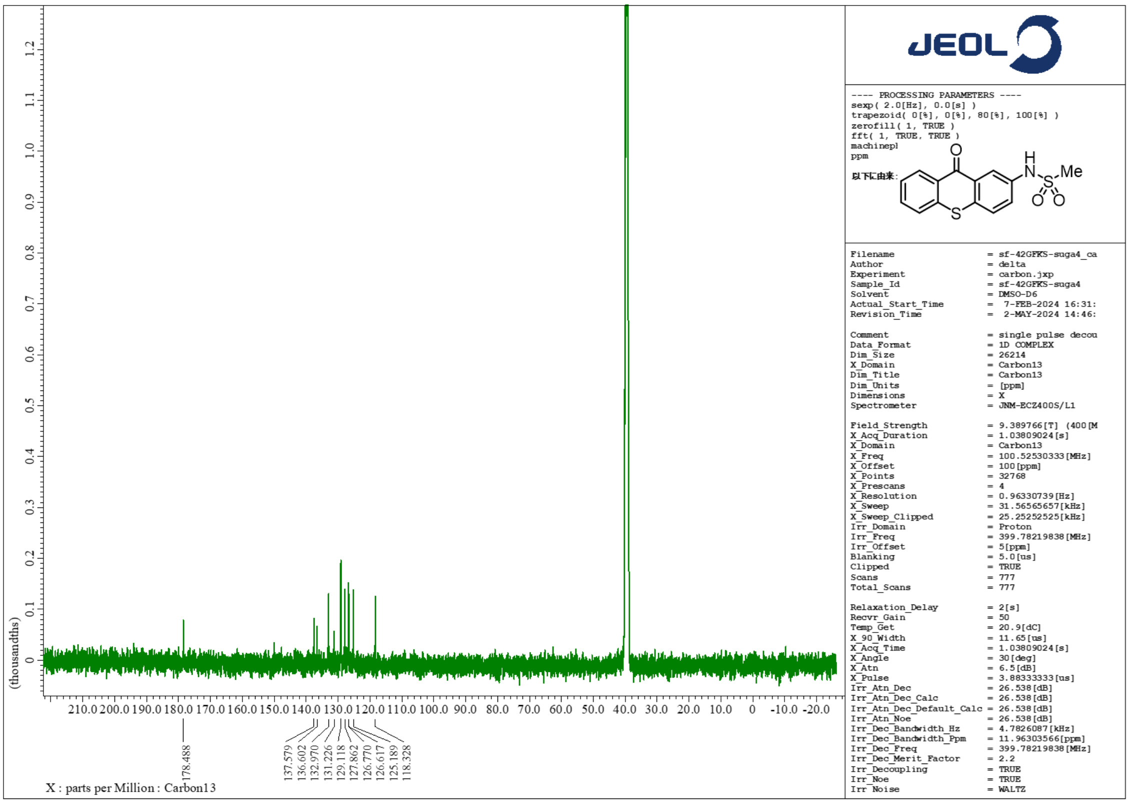

^{1}H-^{1}H COSY NMR (400 MHz, DMSO-d_6) **4**

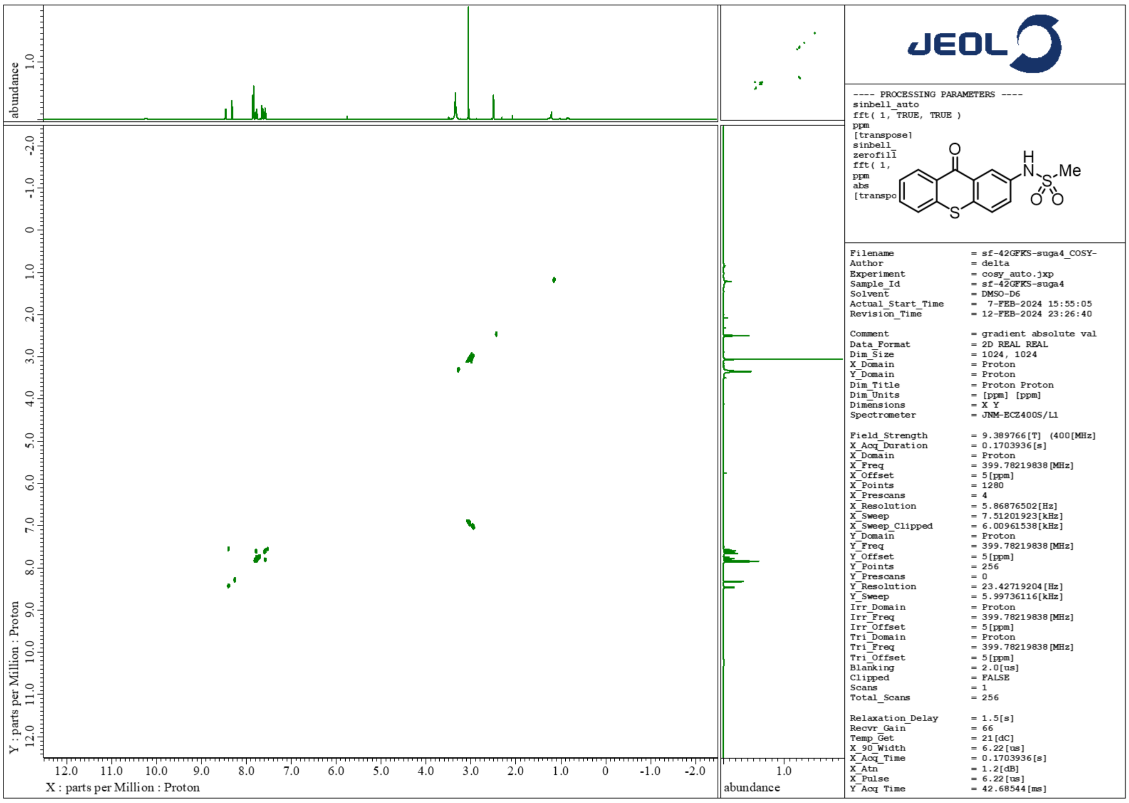

^{1}H-^{13}C HMQC NMR (DMSO-d_6) **4**

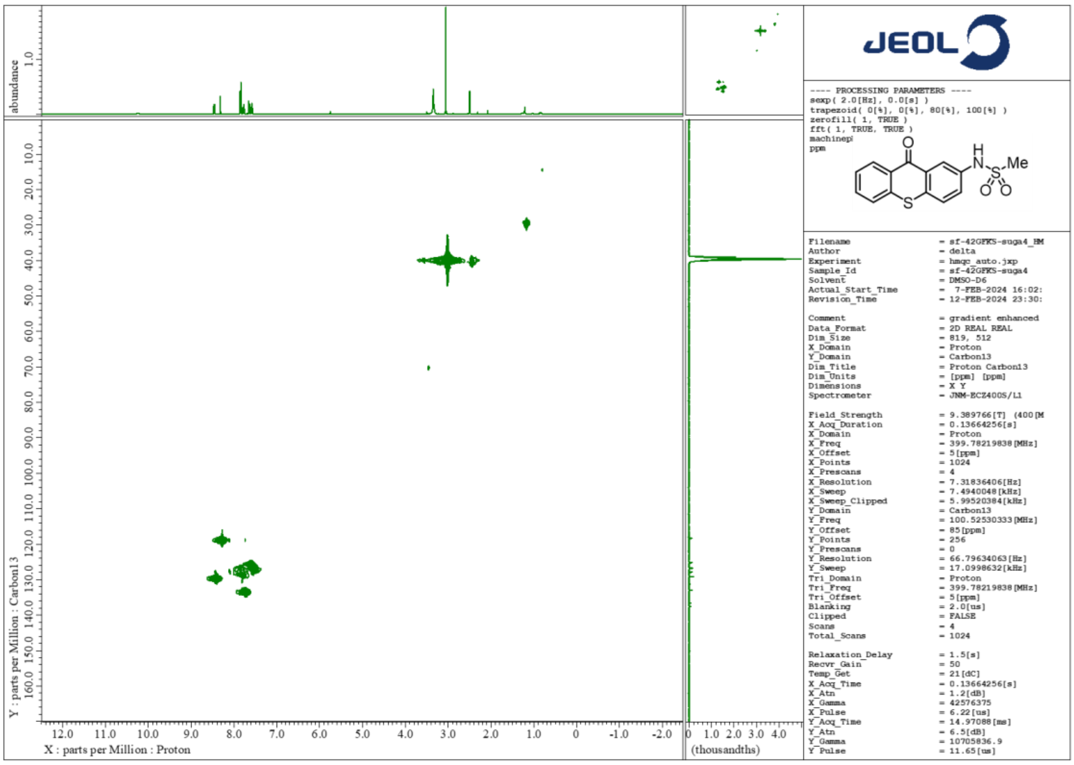

^{1}H NMR (400 MHz, DMSO-d_6) **6a**

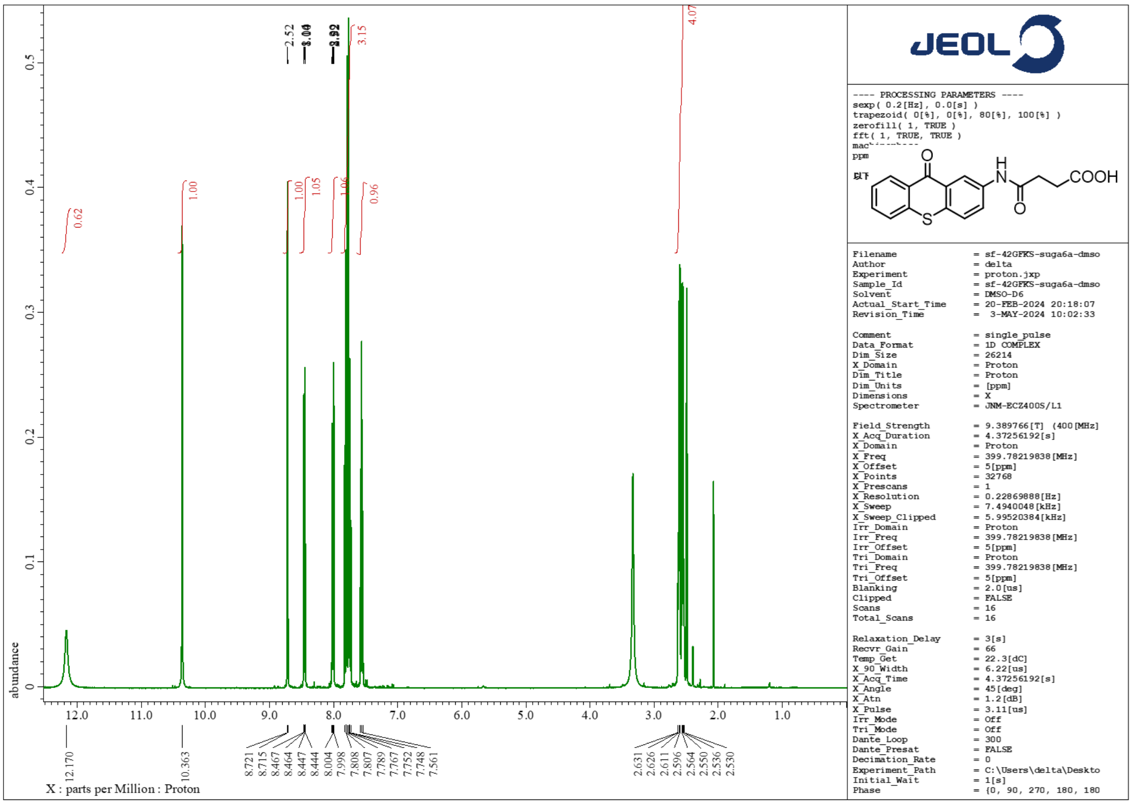

$^{13}C\{^{1}H\}$ NMR (150 MHz, DMSO-d_6) **6a**

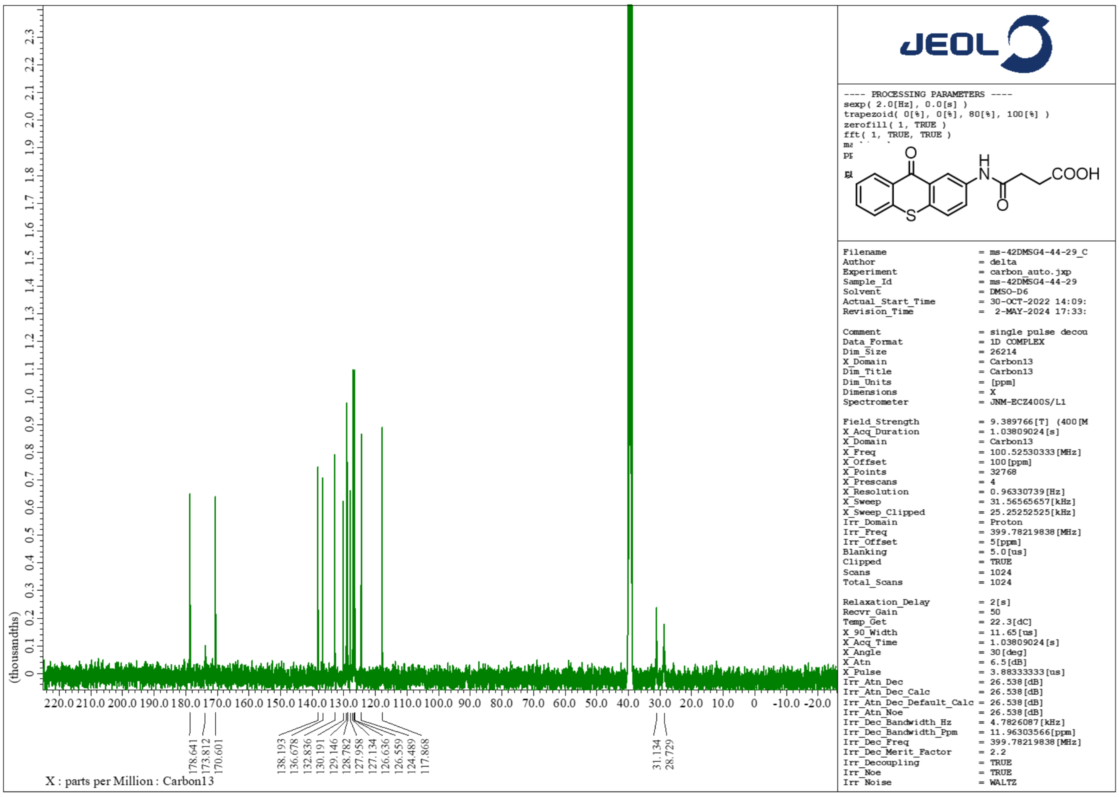

^{1}H-^{1}H COSY NMR (400 MHz, DMSO-*d6*) **6a**

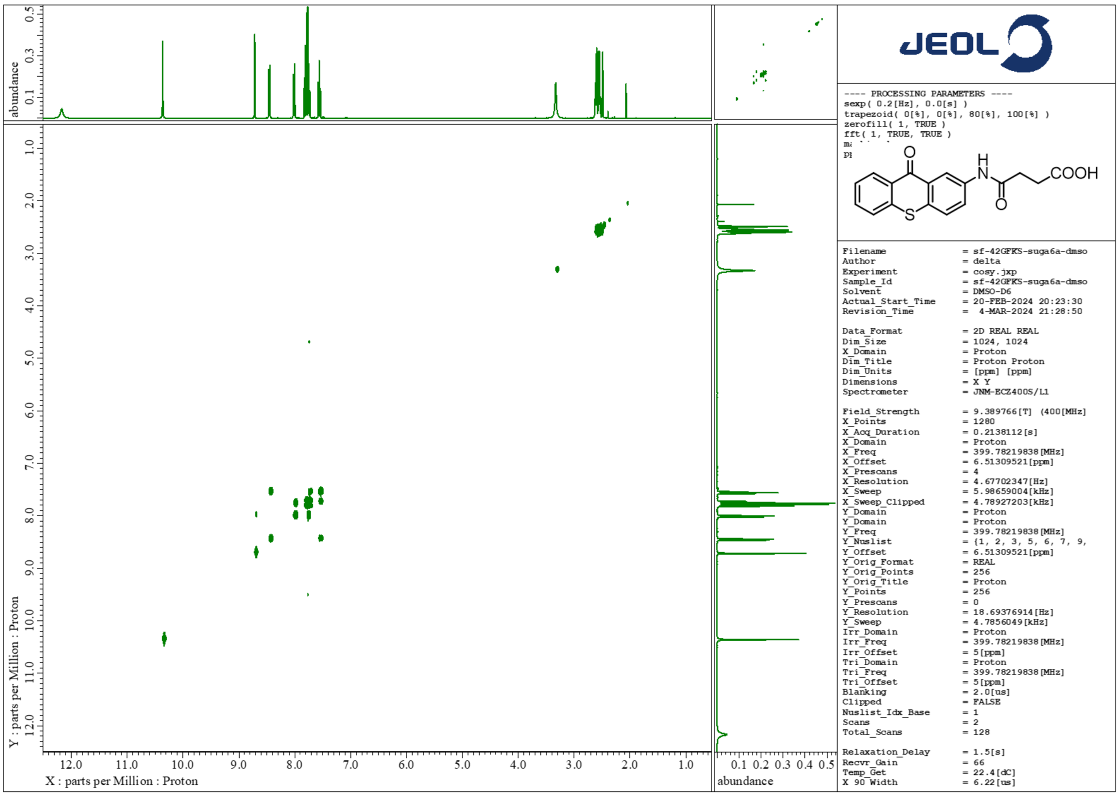

^{1}H-^{13}C HMQC NMR (DMSO-d_6) **6a**

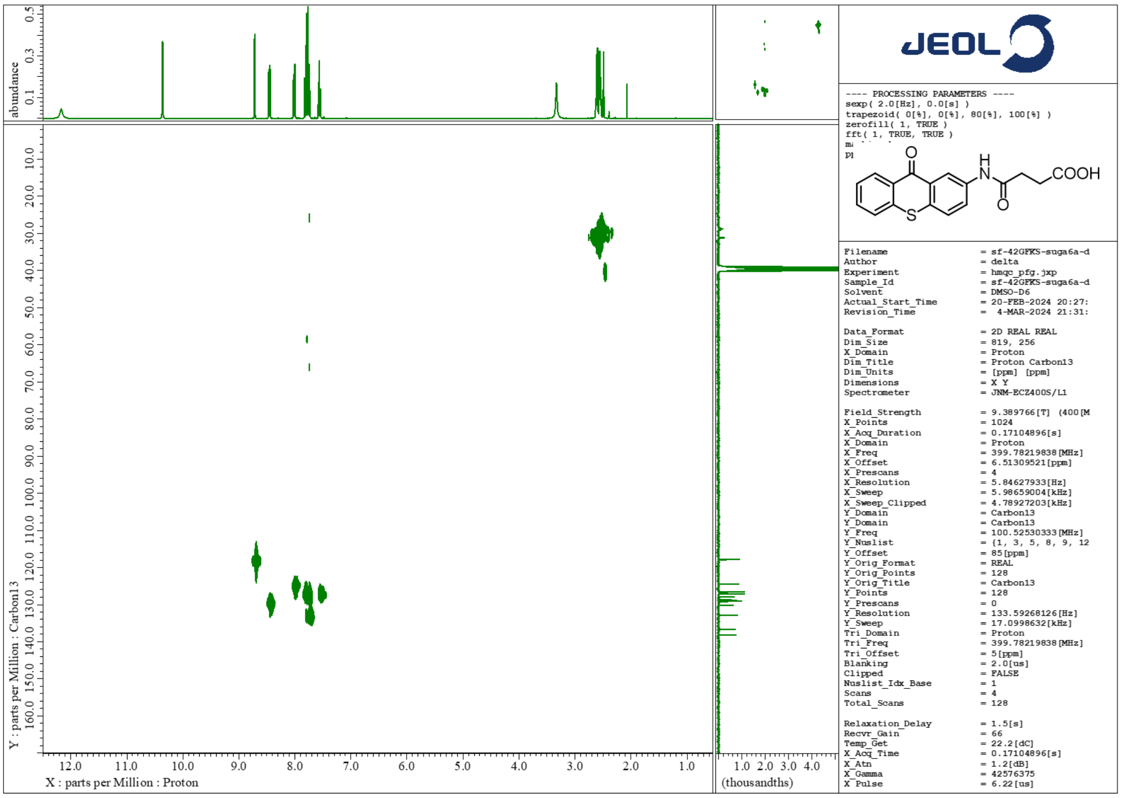

^{1}H NMR (400 MHz, $CDCl_3$) **6b**

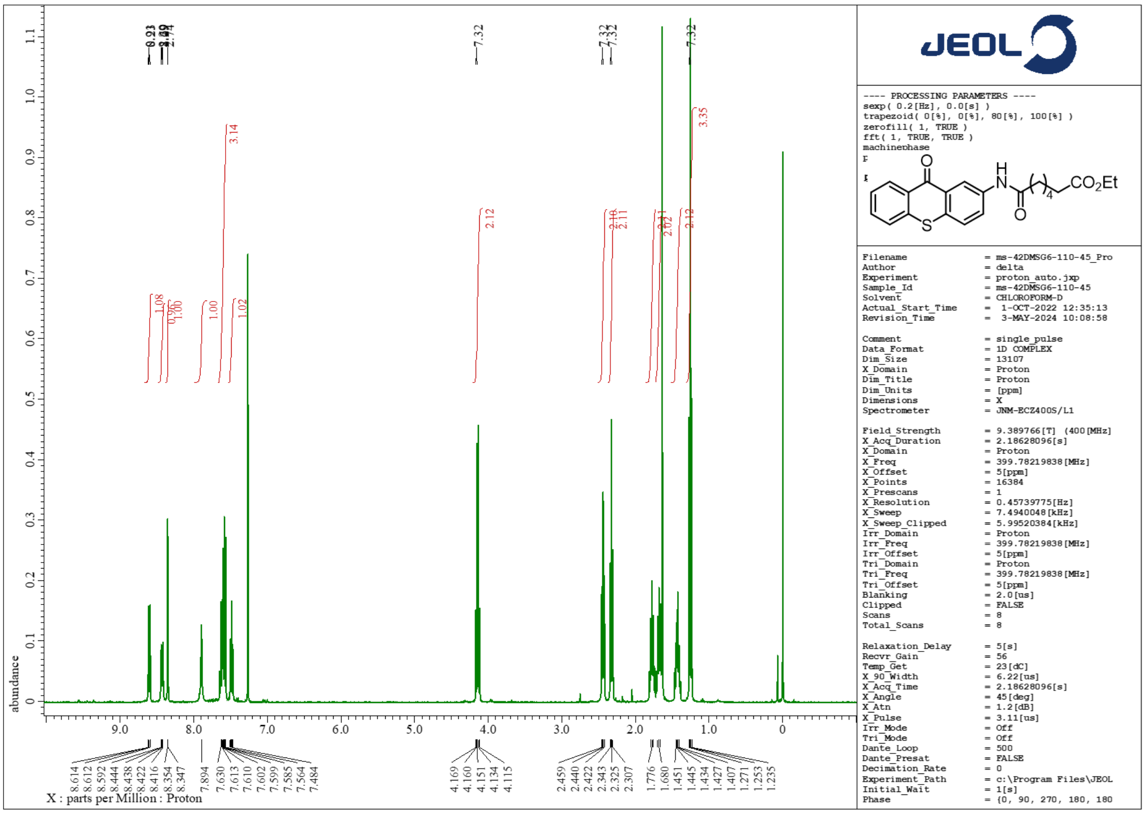

$^{13}C\{^{1}H\}$ NMR (150 MHz, $CDCl_3$) **6b**

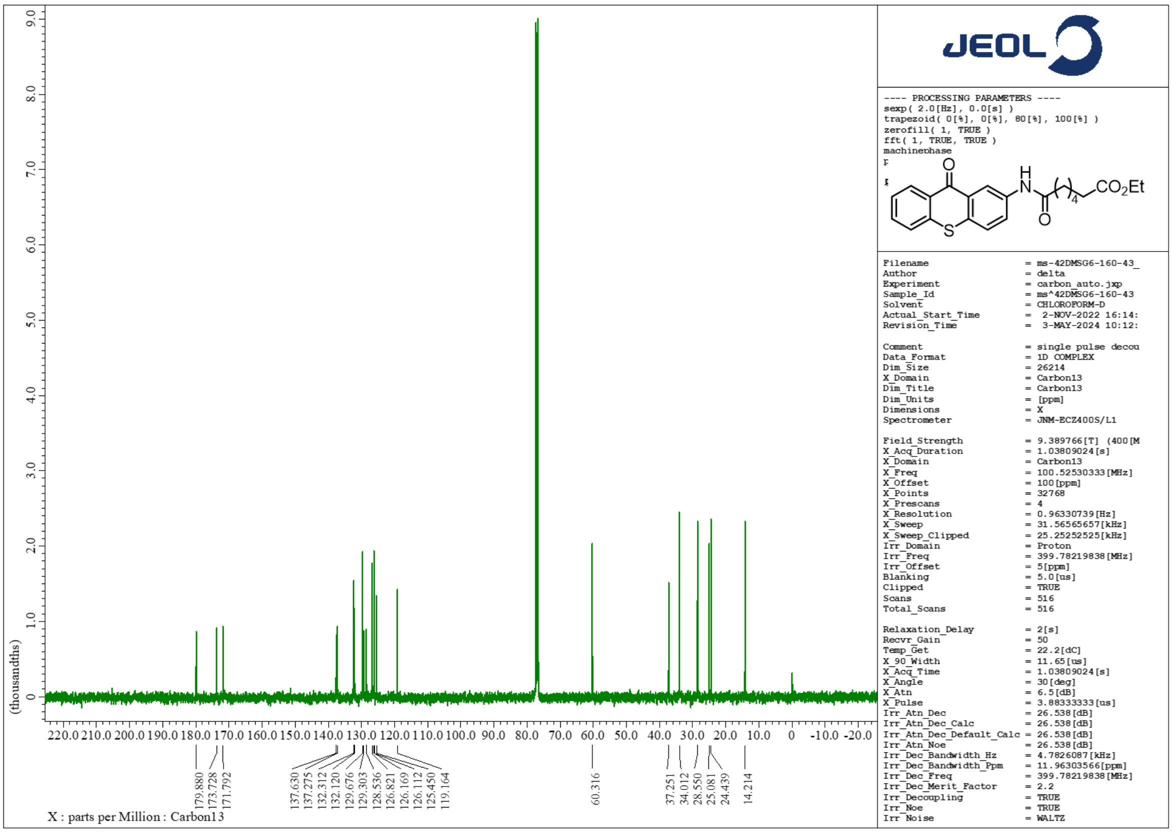

^{1}H-^{1}H COSY NMR (400 MHz, $CDCl_3$) **6b**

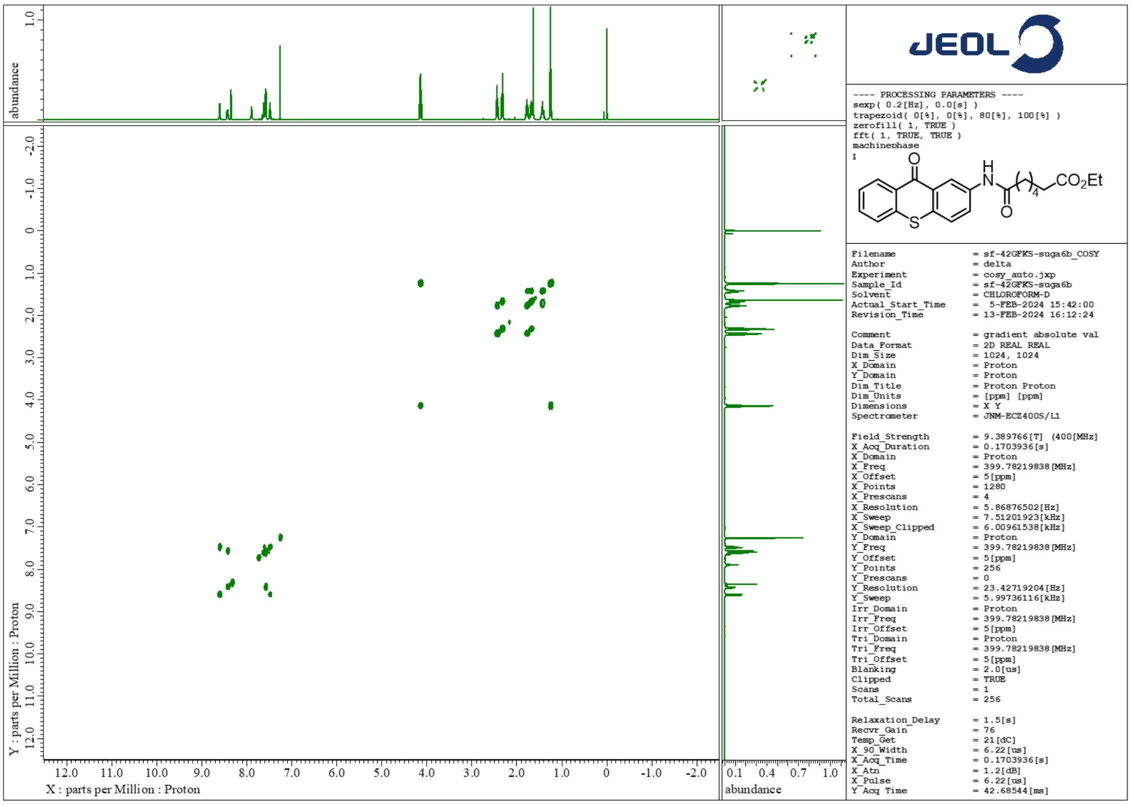

^{1}H-^{13}C HMQC NMR ($CDCl_3$) **6b**

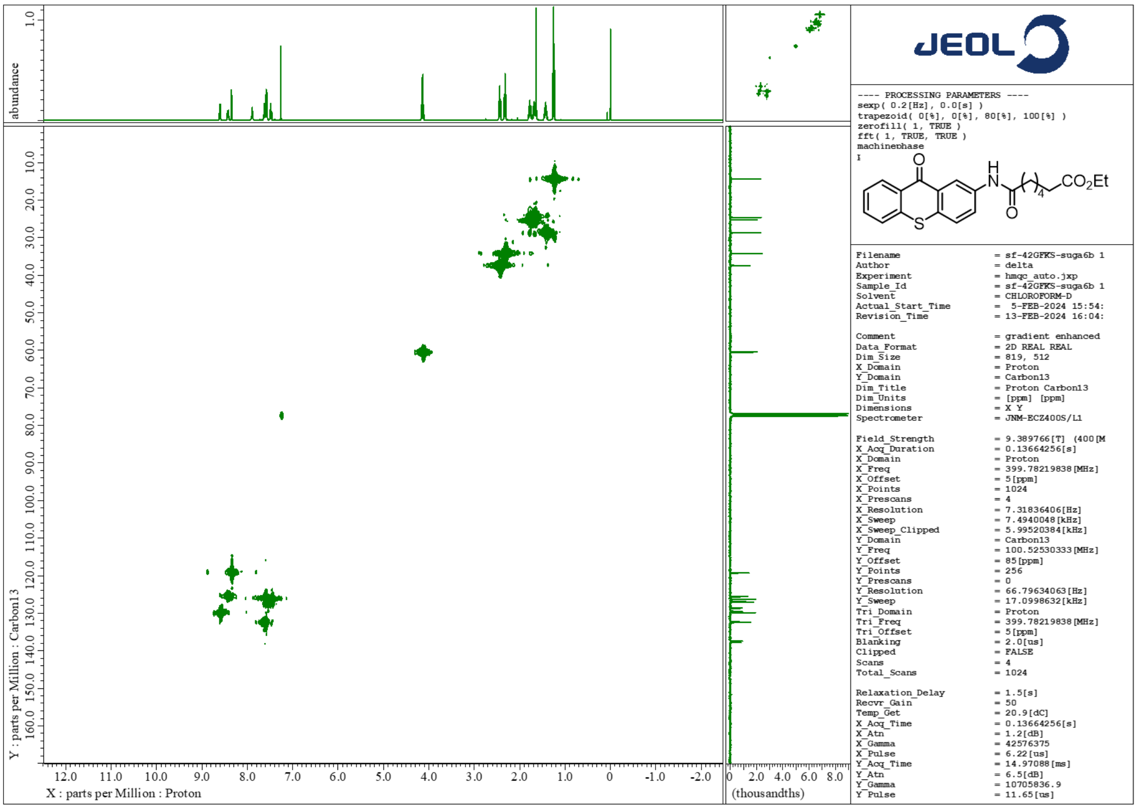

^{1}H NMR (400 MHz, DMSO-d_6) **6c**

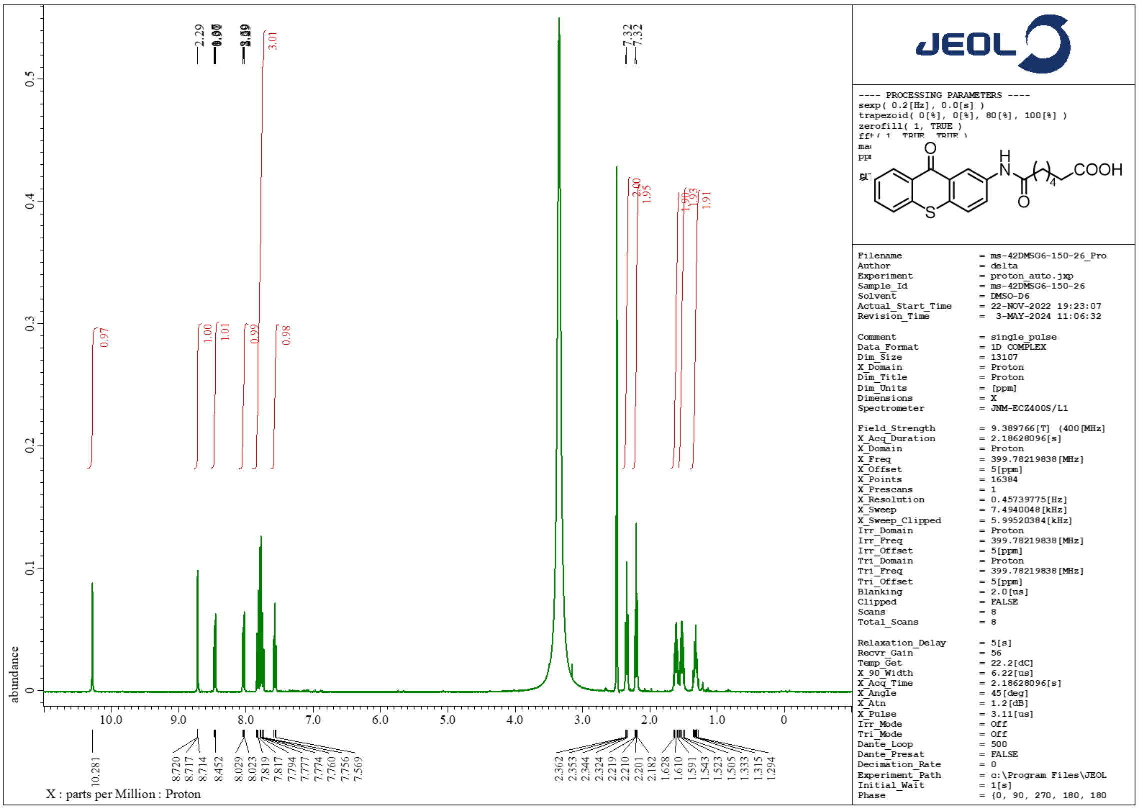

$^{13}C\{^{1}H\}$ NMR (150 MHz, DMSO-d_6) **6c**

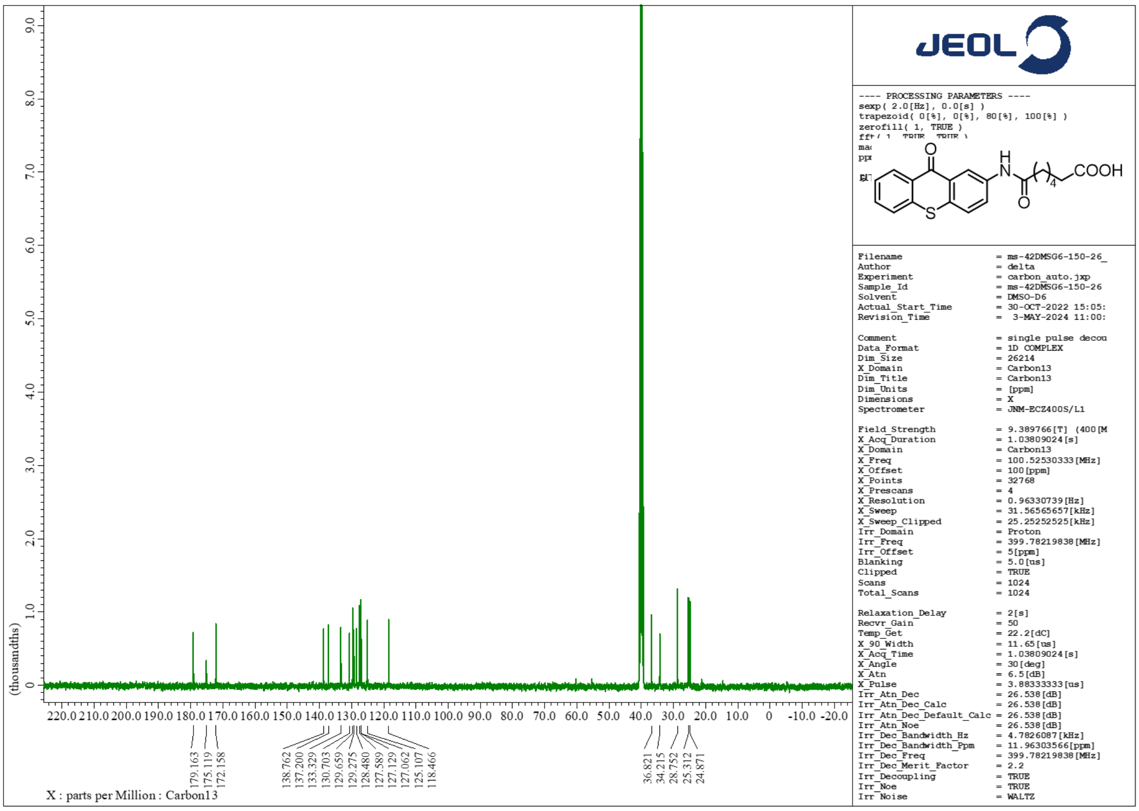

^{1}H-^{1}H COSY NMR (400 MHz, DMSO-d_6) **6c**

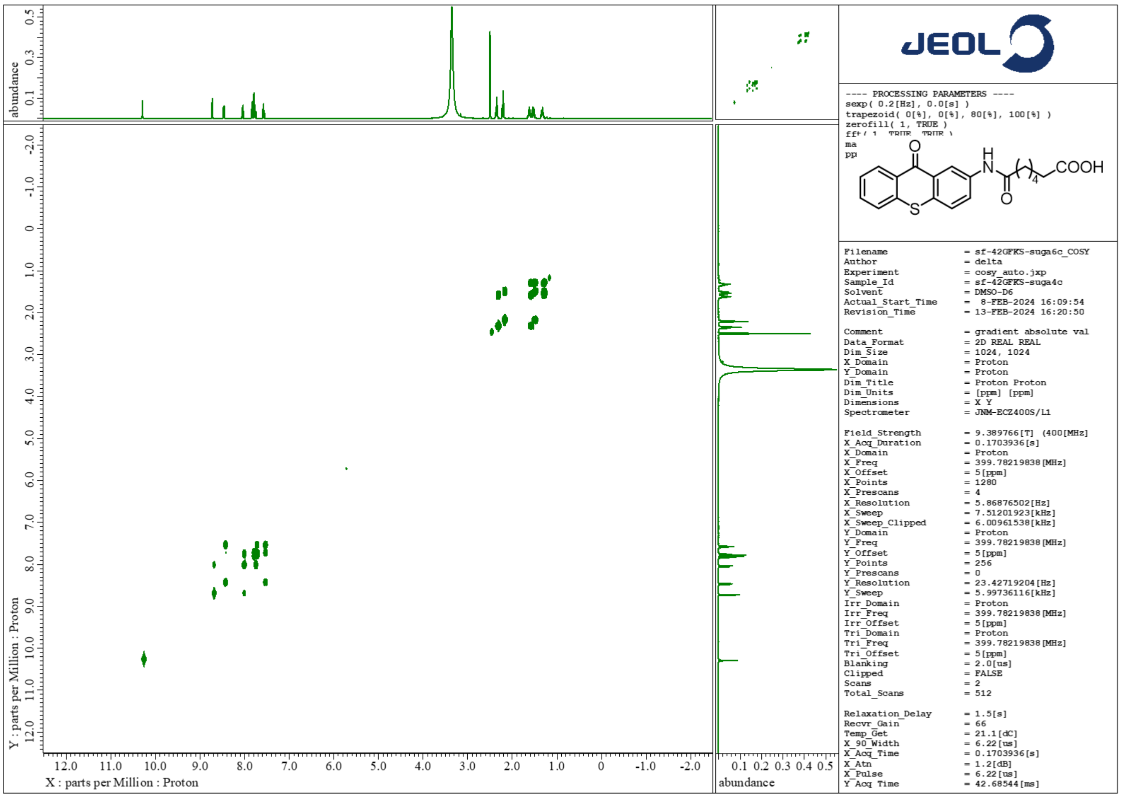

^{1}H-^{13}C HMQC NMR (DMSO-d_6) **6c**

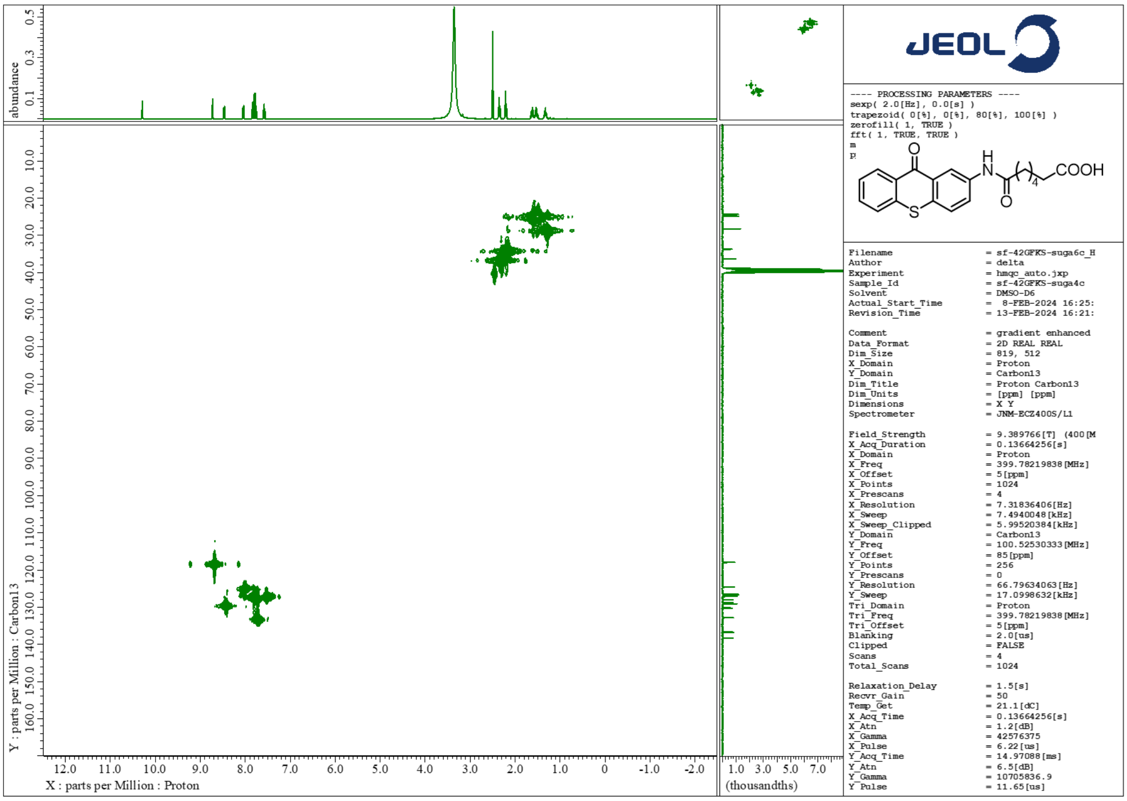

^{1}H NMR (400 MHz, $CDCl_3$) **6d**

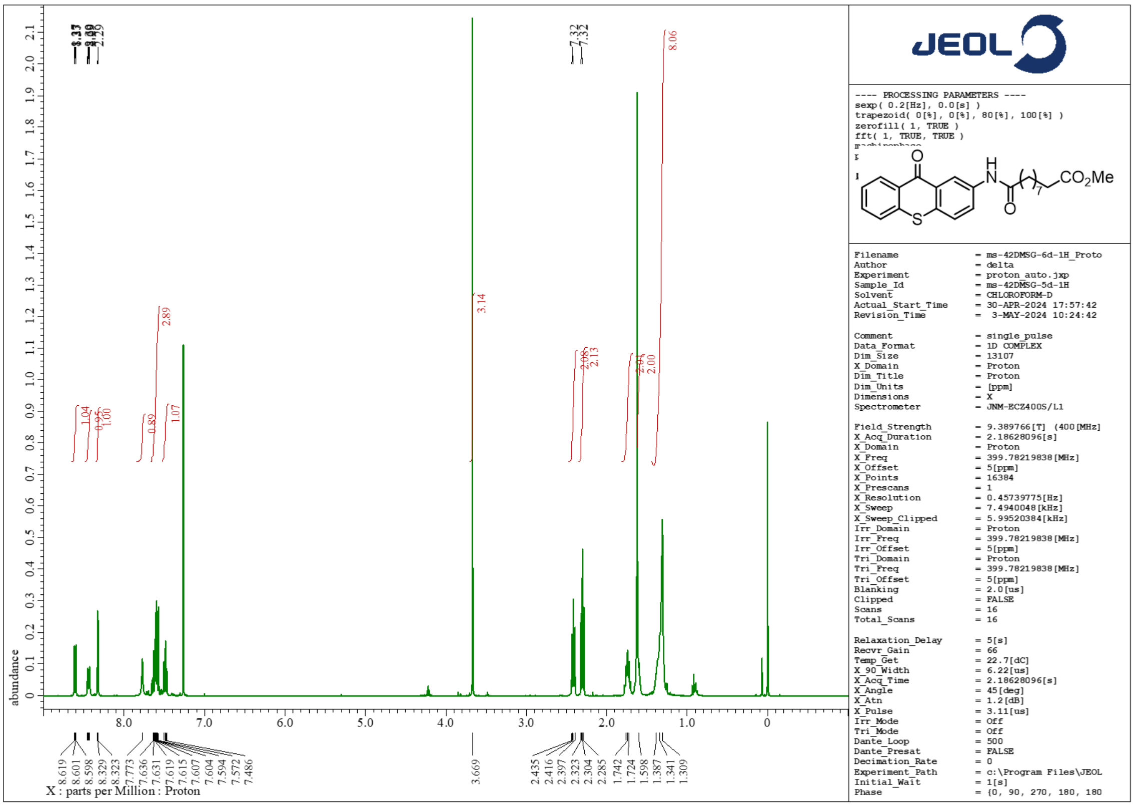

$^{13}C\{^{1}H\}$ NMR (150 MHz, $CDCl_3$) **6d**

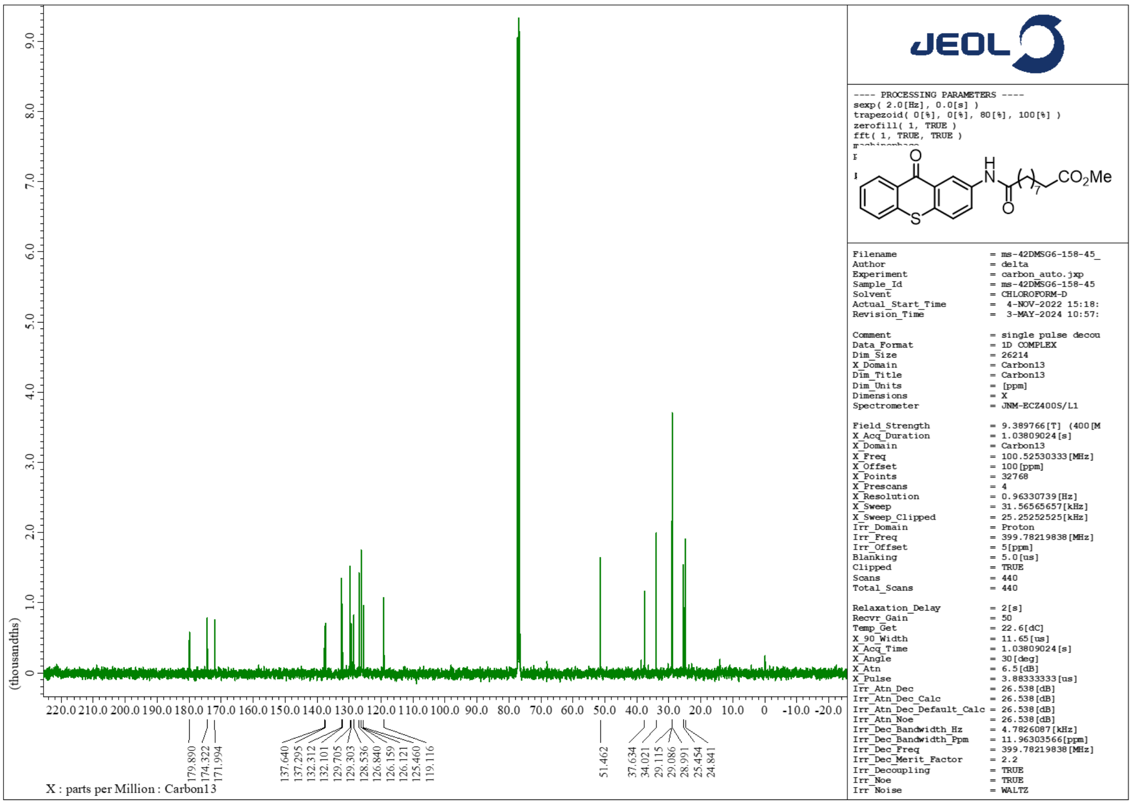

^{1}H-^{1}H COSY NMR (400 MHz, $CDCl_3$) **6d**

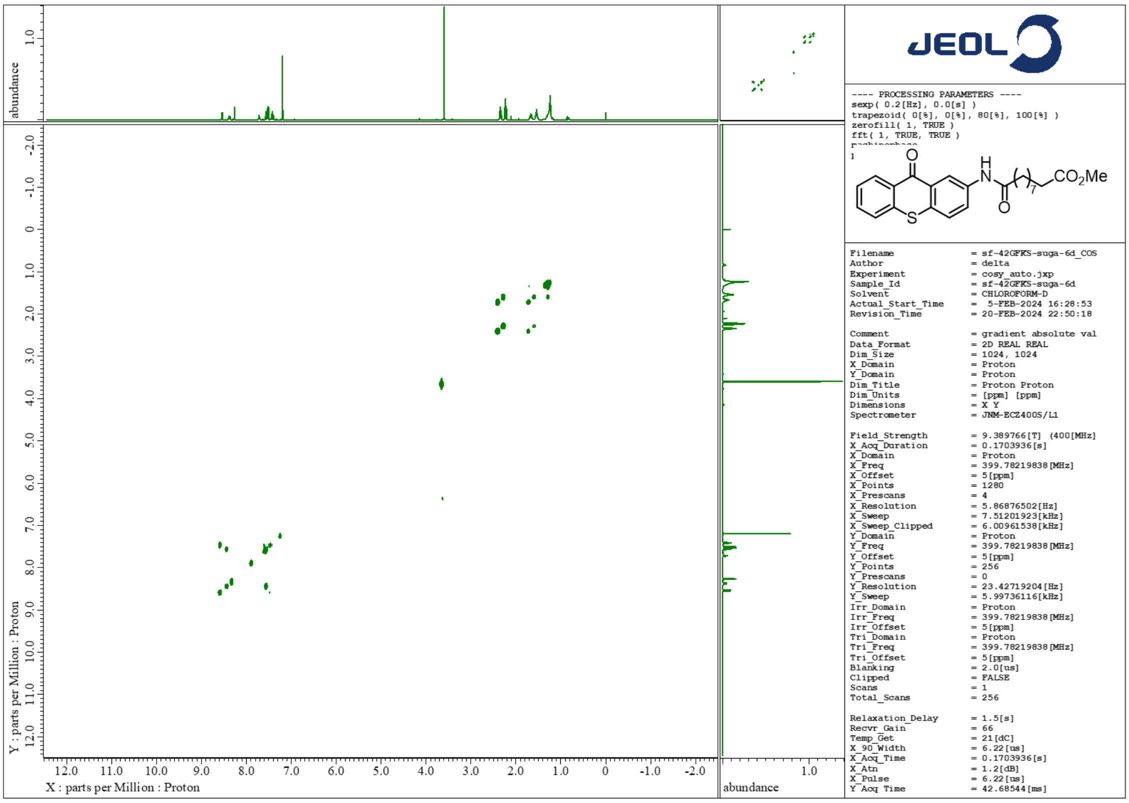

^{1}H-^{13}C HMQC NMR ($CDCl_3$) **6d**

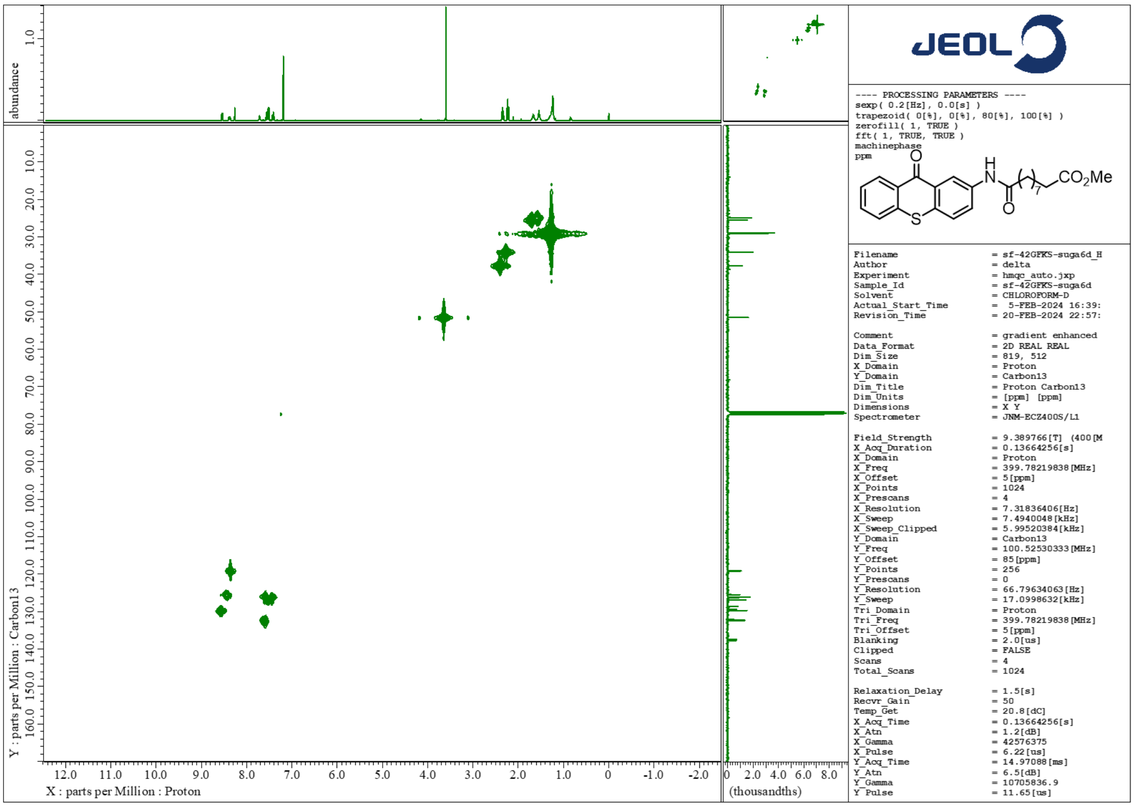

^{1}H NMR (400 MHz, DMSO-d_6) **6e**

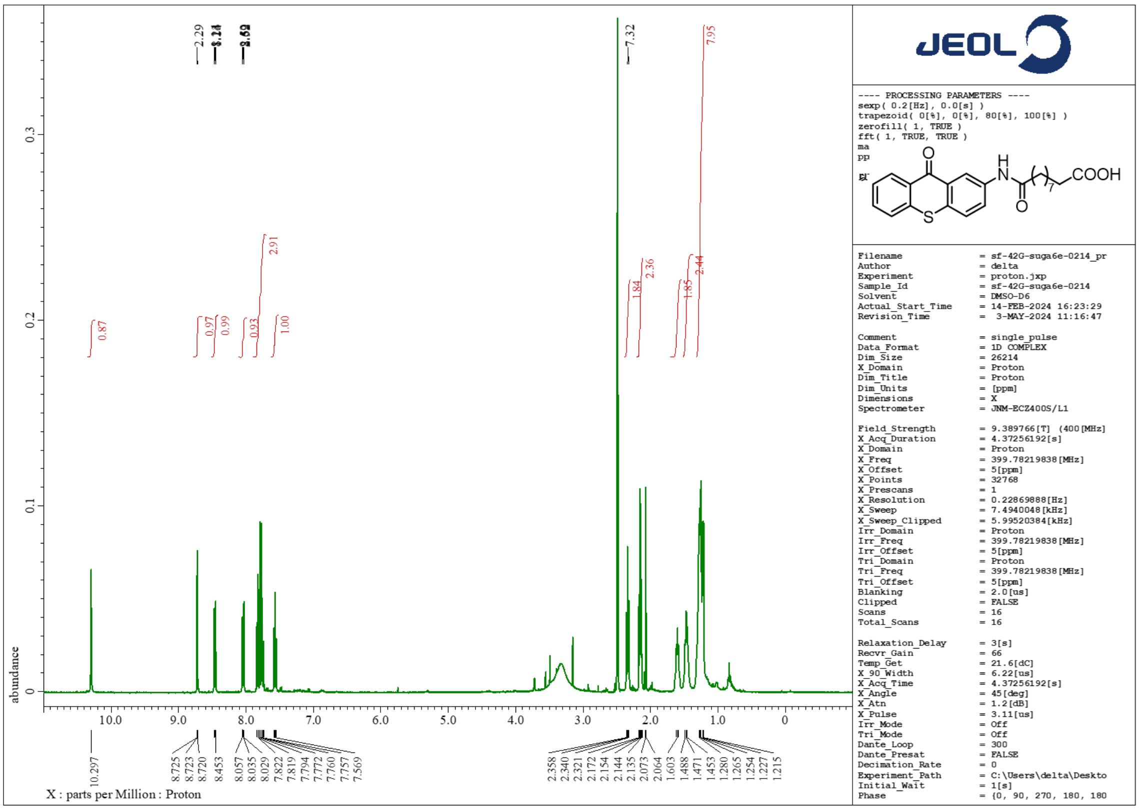

$^{13}C\{^{1}H\}$ NMR (150 MHz, DMSO-d_6) **6e**

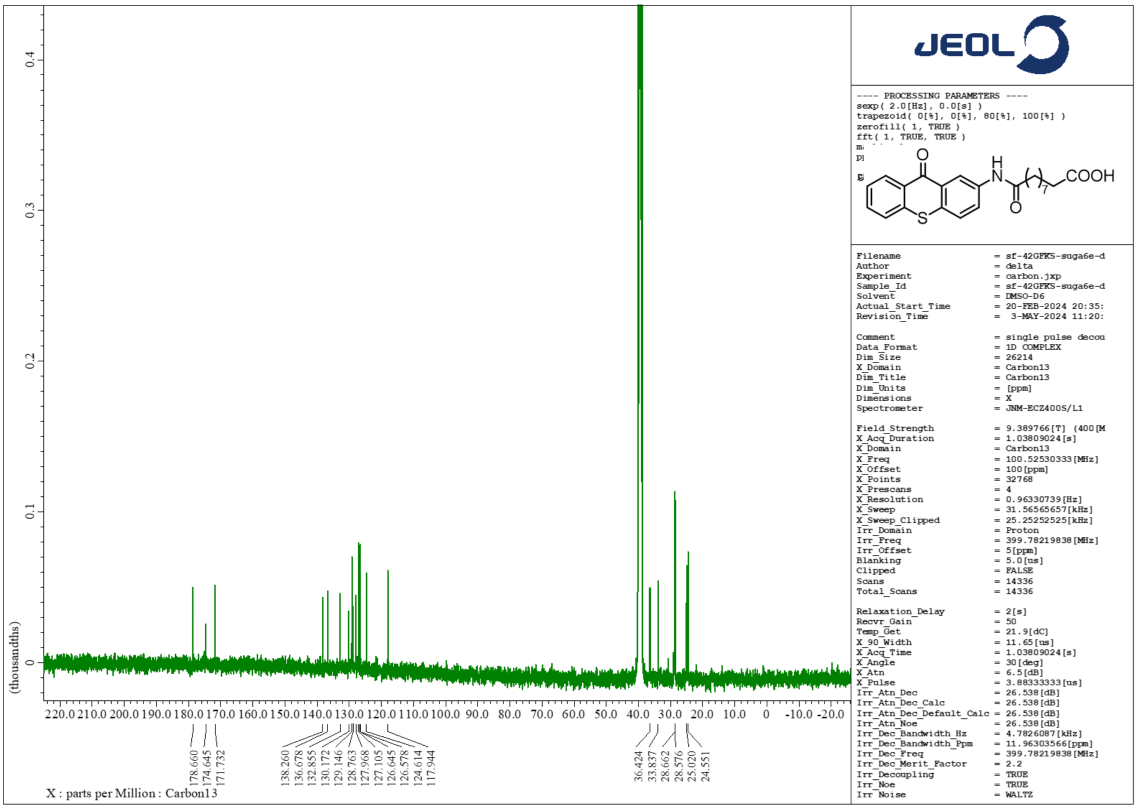

^{1}H-^{1}H COSY NMR (400 MHz, DMSO-d_6) **6e**

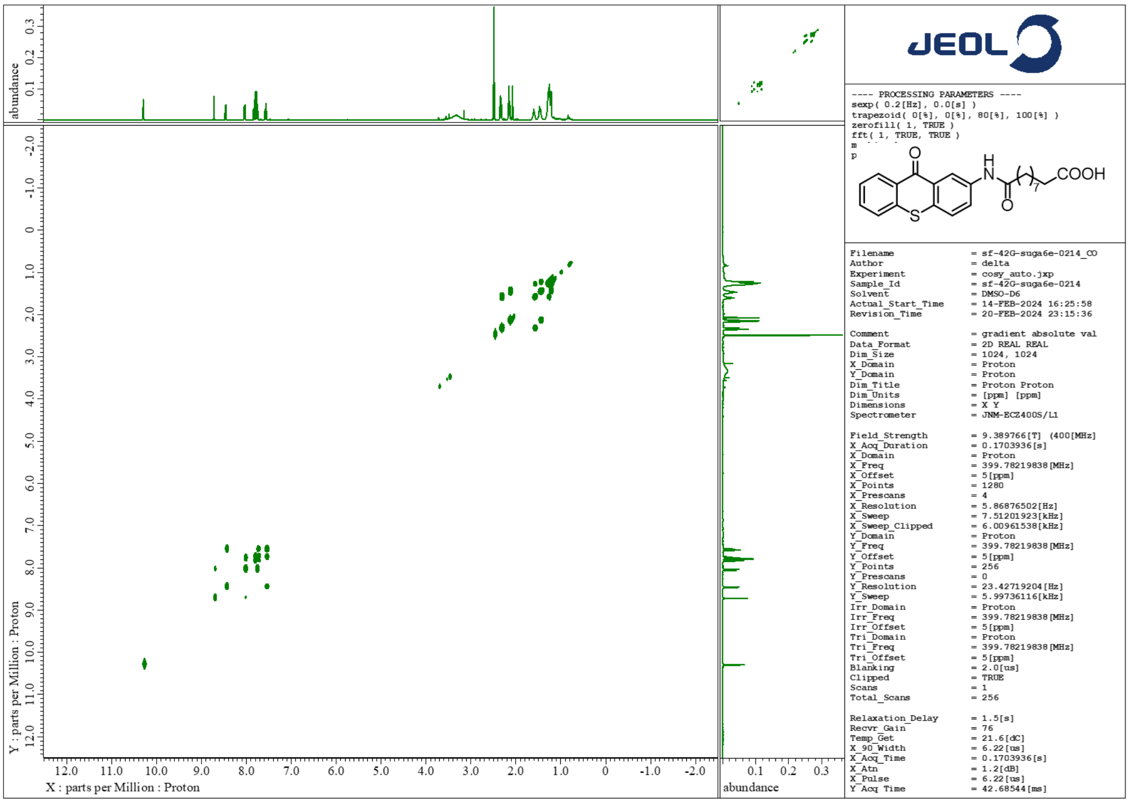

^{1}H-^{13}C HMQC NMR (DMSO-d_6) **6e**

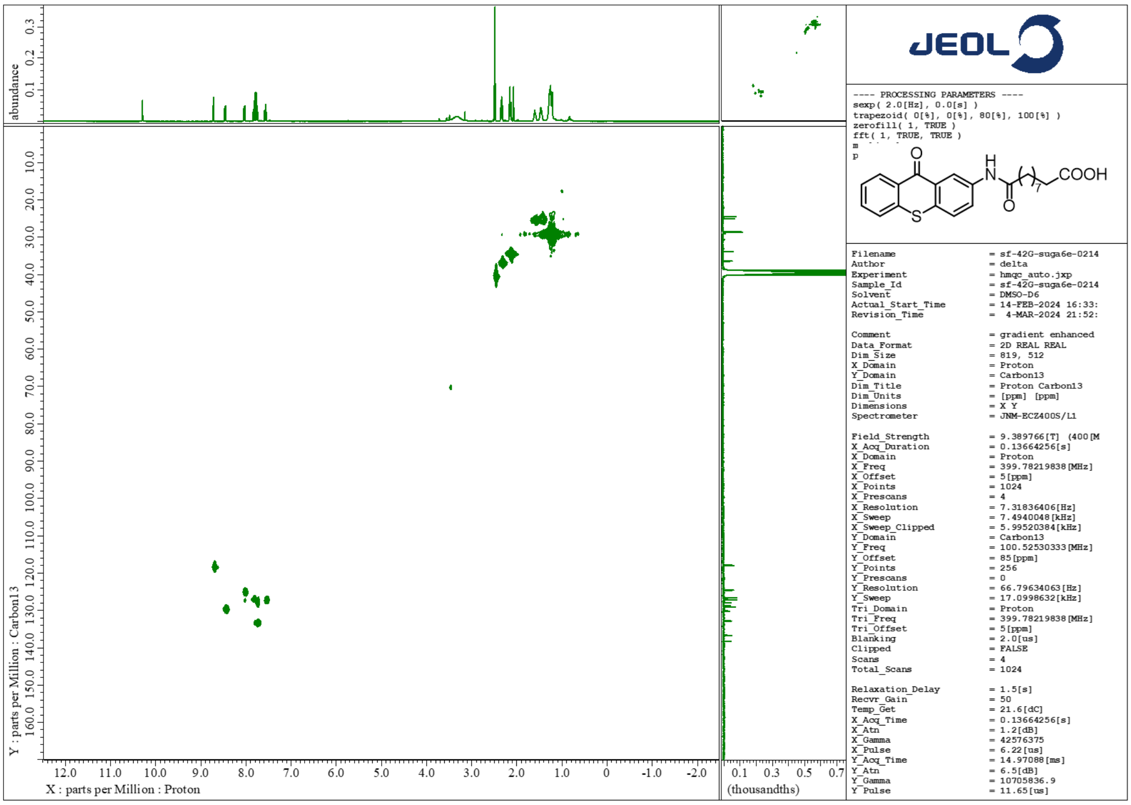

Supplement: Supplementary file 1 — jo4c00721_si_001.pdf [file jo4c00721_si_001.pdf]
